# Exploration and Characterization of the Antimalarial Activity of Pyrimidine‐2,4‐Diamines for which Resistance is Mediated by the ABCI3 Transporter

**DOI:** 10.1002/cmdc.202500739

**Published:** 2025-12-12

**Authors:** Mahta Mansouri, Madeline G. Dans, Zijun Low, Katie Loi, Kate E. Jarman, Jocelyn S. Penington, Deyun Qiu, Adele M. Lehane, Benigno Crespo, Franciso‐Javier Gamo, Delphine Baud, Stephen Brand, Paul F. Jackson, Alan F. Cowman, Brad E. Sleebs

**Affiliations:** ^1^ The Walter and Eliza Hall Institute of Medical Research Parkville 3052 Australia; ^2^ Department of Medical Biology The University of Melbourne Parkville 3010 Australia; ^3^ Research School of Biology Australian National University Canberra 2601 Australia; ^4^ Global Health Medicines R &D, GSK Tres Cantos 28760 Spain; ^5^ MMV Medicines for Malaria Venture 1215 Geneva Switzerland; ^6^ Emerging Science & Innovation, Discovery Sciences Janssen R&D LLC La Jolla CA 92121 USA

**Keywords:** ABCI3 transporter, antimalarial, malaria, *Plasmodium*, pyrimidine‐2,4‐diamine

## Abstract

The spread of drug‐resistant *Plasmodium* strains is diminishing the effectiveness of current antimalarials, highlighting the importance of discovering new therapeutics with novel targets. A screen of the Jumpstarter library against *P. falciparum* identified W482 with a pyrimidine‐2,4‐diamine scaffold. Structure‐activity relationships reveal the importance of the pyrimidine core and its endocyclic nitrogen, while alternative amines are tolerated in the 4‐position. Bulky and hydrophobic carboxamides or substituted phenyl ureas display the most potent antiplasmodial activity. Resistance selection and whole genome sequencing reveal an amplification of the gene encoding the ABCI3 transporter protein W482‐resistant parasites. W482 is found to exhibit greater activity against parasites with reduced expression of ABCI3, confirming that resistance is related to the transporter. W482 arrests asexual parasites at the ring to trophozoite transition stage and exhibits a fast‐killing profile with a lag phase of 24 h. Improving the antiparasitic activity alongside metabolic stability and solubility remains a challenge in the future development of the pyrimidine‐2,4‐diamine class.

## Graphical abstract

Investigation of the structure‐activity relationship on W482 (**1**) with a pyrimidine‐2,4‐diamine scaffold culminated in analog **51** with enhanced anti‐plasmodial activity. Forward genetics and phenotypic examination revealed the ABCI3 transporter protein as a putative resistance mechanism. Enhancing physicochemistry and potency are challenges for the future development of this class.

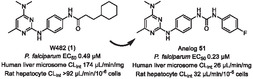

## 1 Introduction

Malaria is a disease caused by *Plasmodium* parasites and transmitted from human to human by female *Anopheles* mosquitoes. It remains a huge burden on health resources, whereby in 2023, 263 million malaria cases were reported worldwide, leading to an estimated 597,000 deaths.^[^ ^1^ ^]^ Countries in Africa account for 95% of these deaths, where children under the age of five are the most vulnerable group.^[^ ^1^ ^]^ Globally, *Plasmodium falciparum* remains the deadliest species and the focus of most drug discovery efforts.

The use of artemisinin‐based combination therapies,^[^ ^2^ ^]^ insecticides, and most recently vaccines,^[^ ^3^ ^,^ ^4^ ^]^ has dramatically reduced the burden of malaria. However, the emergence and spread of artemisinin partial resistance^[^ ^5^, ^6^ ^–^ ^7^ ^]^ and the threat of resistance to artemisinin partner drugs are a major concern.^[^ ^1^ ^,^ ^8^ ^]^ The development of new small‐molecule therapies with novel modes of action is necessary to overcome these resistance mechanisms.^[^ ^9^ ^,^ ^10^ ^]^ Research and development efforts for new antimalarial drugs have included numerous high‐throughput screening campaigns to identify new hits.^[^ ^10^, ^11^, ^12^ ^–^ ^13^ ^]^

**Figure 1:**
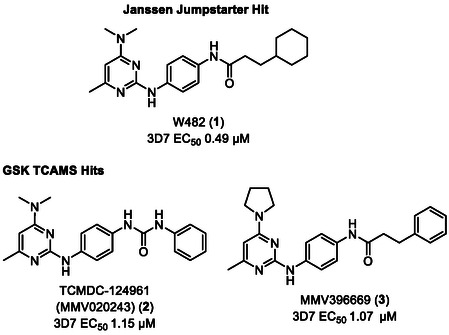
Activity against asexual *P. falciparum* 3D7 parasites of hit compounds with the pyrimidine‐2,4‐diamine scaffold from screens of the Janssen Jumpstarter library and the GSK TCAMS library.

A screen of the Johnson and Johnson Jumpstarter library evaluated 80,000 diverse drug‐like small molecules against *P. falciparum* and identified several hit classes targeting the *P. falciparum* 3D7 asexual blood stage.^[^ ^14^ ^]^ These included 3‐oxadiazole quinolones,^[^ ^14^ ^]^ triazolopyrimidines,^[^ ^15^ ^]^ dihydroquinazolinone‐3‐carboxamides,^[^ ^16^, ^17^ ^–^ ^18^ ^]^ pyrazolopyridine 4‐carboxamides,^[^ ^19^ ^]^ aryl amino acetamides,^[^ ^20^ ^,^ ^21^ ^]^ cyclopropyl carboxamides,^[^ ^22^ ^]^ and N‐acetamide indoles.^[^ ^23^ ^]^ One of the other hits identified in this screen was W482 (**1**) (**Figure** **1**). W482 (**1**) has modest activity against *P. falciparum* asexual parasites (EC_50_ 0.49 µM) and no cytotoxicity against human HepG2 cells (CC_50_ > 40 µM). The structure of this hit class is comprised of a pyrimidine‐2,4‐diamine motif connected to a central phenyl ring substituted in the 4‐position with an aliphatic substituted carboxamide. This structural class (compounds **2** and **3**) was also identified by GSK from their screen of the Tres Cantos Antimalarial Set (TCAMS)^[^ ^11^ ^,^ ^24^ ^]^ and was subsequently included in the MMV Malaria Box (Figure 1).^[^ ^25^ ^]^ To the best of our knowledge, there has been no exploration of the structure–activity relationship (SAR) or characterization of the antiplasmodial activity of the pyrimidine‐2,4‐diamine class, and therefore, we sought to investigate the potential of this structural class for antimalarial development.

In this research, we explored the SAR of the pyrimidine‐2,4‐diamine chemotype against the *P. falciparum* asexual stage parasite while monitoring human HepG2 cytotoxicity and assessing physicochemical properties. In parallel to the SAR study, to understand the mechanism by which this compound class kills the parasite, we selected for parasites resistant to W482 (**1**). Whole genome sequencing of W482‐resistant parasites uncovered an amplification of the region in the genome that encodes the ABCI3 transporter. To provide further evidence that parasite susceptibility to W482 is associated with the ABCI3 transporter, we evaluated W482 (**1**) against genetically engineered parasites in which the ABCI3 transporter can be knocked down. Finally, we characterized the asexual stage activity of W482 (**1**). Collectively, this research aims to assess the suitability of the pyrimidine‐2,4‐diamine class for antimalarial development.

## 2 Results and Discussion

### 2.1 Structure‐Activity Relationship

To establish the SAR, the antiparasitic activity of the analogs was determined using an assay that measures the lactate dehydrogenase (LDH) activity of asexual *P. falciparum* 3D7 parasites after 72 h of compound treatment. Mammalian cytotoxicity was monitored using an assay that measures the growth of HepG2 cells using Cell Titer‐Glo after 48 h of compound treatment.

Investigation into the SAR began with exploring the importance of the substituted amine in the 4‐position of the pyrimidine core. It was found that analog **4** with a 4‐diethylamine substituent was equipotent (EC_50_ 0.52 µM) compared to W482 (**1**) with a 4‐dimethylamine substituent (EC_50_ 0.51 µM) (**Table** **1**). This suggested that there may be room to expand the steric volume of the substituent in this position. Analogs **5** and **6** with 4‐ethylmethylamine and 4‐benzylmethylamine substitution did not have greater antiparasitic activity (EC_50_ 0.51 µM and 0.60 µM) compared to W482 (**1**). Derivatives **7** and **8** with *N*‐pyrrolidine and *N*‐piperidine substitution also exhibited similar antiplasmodial activity (EC_50_ 0.54 µM and 0.43 µM), suggesting that there is no advantage to incorporating groups with added molecular weight and lipophilicity. Polar groups that could aid in solubility were also trialed. For example, analog **9** with *N*‐morpholine demonstrated a 4‐fold reduction in activity (EC_50_ 1.90 µM), while compounds **10** and **11** with *N*‐piperazine and *N*‐methyl *N*‐piperazine exhibited antiparasitic activity (EC_50_ 0.39 µM and 0.47 µM) that is comparable to the activity shown by W482 (**1**). Among these analogs, **4**, **7,** and **8** that are more lipophilic (cLogP > 5) than **1**, showed modest toxicity against human HepG2 cells (CC_50_ 12.6 µM and 9.1 µM), while other analogs were not cytotoxic (CC_50_ > 20 µM). Altogether, these data demonstrate that varying the substitution in the 4‐position of the pyrimidine does not significantly influence the antiparasitic activity, suggesting that the moiety in the 4‐position is oriented into a large cavity of the molecular target.

**Table 1:** Activity of 4‐substituted amine analogs.

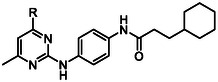				
Compound	R	Pf parasite EC_50_ (SD) [μM]a)	HepG2 CC_50_ (SD) [μM]b)	cLogPc)
W482 (**1**)	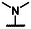	0.49 (0.20)	>20	4.6
**4**	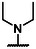	0.52 (0.09)	18.0 (0.7)	5.4
**5**	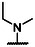	0.51 (0.08)	>20	5.0
**6**	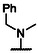	0.60 (0.03)	–	6.0
**7**	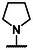	0.54 (0.08)	12.6 (0.6)	5.2
**8**	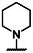	0.43 (0.02)	9.1 (1.0)	5.5
**9**	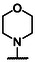	1.90 (0.03)	>20	4.4
**10**	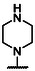	0.42 (0.06)	>20	4.2
**11**	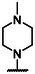	0.47 (0.06)	>20	4.5

a) EC_50_ data represent means and SDs for 3 or more experiments measuring LDH activity of *P. falciparum* 3D7 parasites following 72 h exposure to compounds.

b) CC_50_ data represent means and SDs for 3 experiments measuring HepG2 viability over 48 h using Cell Titer‐Glo.

c) cLogP was calculated using DataWarrior.^[^ ^34^ ^]^

We then investigated the effect of substitution in the 5‐ and 6‐positions on the pyrimidine core while maintaining the 4‐dimethylamine substitution. Analog **12**, in which the 6‐methyl substituent was deleted, did not differ in its antiparasitic activity (EC_50_ 0.57 µM) compared to W482 (**1**), while analogs **13** and **14** with 6‐trifluoromethyl and 6‐methoxy substitution, spanning both polar electron‐donating and lipophilic electron‐withdrawing substituents, were inactive (EC_50_ > 10 µM) (**Table** **2**). 5,6‐Fused ring systems were also synthesized and demonstrated that cyclic aliphatic variant **15** had reduced activity (EC_50_ 1.11 µM) compared to W482 (**1**), while the conjugated derivative **16** slightly improved activity (EC_50_ 0.30 µM), although its cLogP was increased compared to W482 (**1**) (5.4 versus 4.6). This increase in lipophilicity could be attributed to the modest increase in human HepG2 cell cytotoxicity (CC_50_ 14.0 and 11.2 µM for **15** and **16**). These data demonstrated that a larger substituent than a methyl group in the 6‐position was tolerated, whereas electron‐donating or withdrawing substituents were deleterious to antiparasitic activity.

**Table 2:** Activity of analogs with 5‐ and 6‐substituent modification.

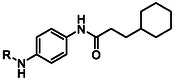				
Compound	R	Pf parasite EC_50_ (SD) [µM]a)	HepG2 CC_50_ (SD) [µM]b)	cLogPc)
W482 (**1**)	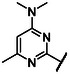	0.49 (0.20)	>20	4.6
**12**	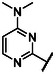	0.57 (0.05)	>20	4.2
**13**	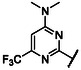	>10	–	4.5
**14**	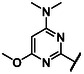	>10	–	5.1
**15**	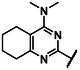	1.11 (0.12)	14.0 (1.6)	5.4
**16**	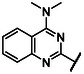	0.30 (0.02)	11.2 (0.3)	5.5

a) EC_50_ data represent means and SDs for 3 or more experiments measuring LDH activity of *P. falciparum* 3D7 parasites following 72 h exposure to compounds.

b) CC_50_ data represent means and SDs for 3 experiments measuring HepG2 viability over 48 h using Cell Titer‐Glo.

c) cLogP was calculated using DataWarrior.^[^ ^34^ ^]^

Iterations to the pyrimidine core were then trialed to determine the impact on antiplasmodial activity of relocating an endocyclic nitrogen to another position or replacing an endocyclic nitrogen with a carbon. Analogs **17** and **18**, whereby the endocyclic nitrogen configuration was altered, both exhibited a decrease in antiplasmodial activity (EC_50_ 5.73 and 1.12 µM, respectively) (**Table** **3**). Furthermore, pyridine analogs **19** and **20** were both less active than the hit pyrimidine (EC_50_ 0.61 and 0.88 µM, compared to EC_50_ 0.49 µM). Due to the desirable activity of quinazoline containing **16**, endocyclic iterations of this core were also trialed. Replacing the endocyclic nitrogen with a carbon was not tolerated in this scaffold either, with both isoquinoline **21** and quinoline **22** showing a reduction in antiplasmodial activity (EC_50_ > 10 and 2.47 µM, respectively). Curiously, the position of the endocyclic nitrogen in the pyridine analog **19** and isoquinoline **21** has vastly different activities, suggesting divergence in SAR and possible target poly‐pharmacology. Altogether, these data suggest that the positioning of the nitrogen atoms in the core scaffold is important for the antiplasmodial activity, and that the 2,4‐endocyclic nitrogen configuration in the hit compound W482 (**1**) and analog **16** is optimal for activity.

**Table 3:** Activity of analogs with alternative heterocycles.

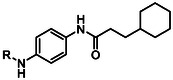				
Compound	R	Pf parasite EC_50_ (SD) [µM]a)	HepG2 CC_50_ (SD) [µM]b)	cLogPc)
W482 (**1**)	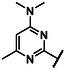	0.49 (0.20)	>20	4.6
**17**	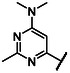	5.73 (0.32)	–	4.4
**18**	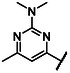	1.12 (0.16)	>20	4.6
**19**	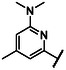	0.61 (0.01)	–	5.1
**20**	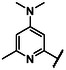	0.81 (0.24)	–	4.8
**16**	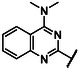	0.30 (0.02)	11.2 (0.3)	5.5
**21**	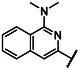	>10	–	5.9
**22**	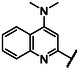	2.48 (0.03)	15.9 (0.9)	5.7

a) EC_50_ data represent means and SDs for 3 or more experiments measuring LDH activity of *P. falciparum* 3D7 parasites following 72 h exposure to compounds.

b) CC_50_ data represent means and SDs for 3 experiments measuring HepG2 viability over 48 h using Cell Titer‐Glo.

c) cLogP was calculated using DataWarrior.^[^ ^34^ ^]^

Fluorine atoms and endocyclic nitrogen are known to influence membrane permeability, solubility, and metabolism. We explored whether fluorine substitution or the inclusion of endocyclic nitrogens in the central aryl ring would affect antiparasitic activity. Compounds **23** and **24** with fluorine in the 2‐ or 3‐positions of the central phenyl ring had a three‐ and two‐fold lower antiplasmodial activity (EC_50_ 1.83 and 1.12 µM) compared to W482 (**1**) (**Table** **4**). Derivative **26** with an endocyclic nitrogen in the 3‐position showed a slight decrease in activity (EC_50_ 0.78 µM) while analog **25** with an endocyclic nitrogen in the 4‐position exhibited an 8‐fold reduction in activity (EC_50_ 3.86 µM). Derivative **26** has a decreased cLogP (4.0), implying that the incorporation of a nitrogen at this position could be useful in future for modulating the physicochemical properties of this compound class. Although the analogs in this subset did not exhibit adverse cytotoxicity against human HepG2 cells (CC_50_ > 20 µM), the inclusion of a fluorine or endocyclic nitrogen was not advantageous for antiparasitic activity, so these iterations were not further explored.

**Table 4:** Activity of analogs with central ring modifications.

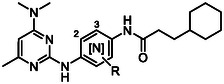				
Compound	X	Pf parasite EC_50_ (SD) [µM]a)	HepG2 CC_50_ (SD) [µM]b)	cLogPc)
W482 (**1**)	Ph	0.49 (0.20)	>20	4.6
**23**	2‐F	1.83 (0.32)	>20	4.7
**24**	3‐F	1.12 (0.06)	>20	4.7
**25**	2‐N	3.86 (0.46)	>20	4.0
**26**	3‐N	0.78 (0.11)	>20	4.0

a) EC_50_ data represent means and SDs for 3 or more experiments measuring LDH activity of *P. falciparum* 3D7 parasites following 72 h exposure to compounds.

b) CC_50_ data represent means and SDs for 3 experiments measuring HepG2 viability over 48 h using Cell Titer‐Glo.

c) cLogP was calculated using DataWarrior.^[^ ^34^ ^]^

The importance of the 3‐cyclohexyl propane substitution on the carboxamide moiety was then explored. This investigation assisted in determining the overlap in activity with the structurally similar hit compounds **2** and **3** from the GSK TCAM screen (Figure 1). Replacement of the terminal aliphatic substituent with 5‐membered aromatic groups resulted in a significant loss in activity, as demonstrated by analogs **27** and **28** with 2‐thiophene and 2‐furan substitution (EC_50_ 1.44 and 3.90 µM) (**Table** **5**), while compound **29** with a 2‐naphthyl variation exhibited similar potency (EC_50_ 0.36 µM) to W482 (**1**). These data inferred that the overall lipophilicity of a large aromatic group (cLogP 5.0) may be contributing to antiparasitic activity. Decreasing the size of the aliphatic substituent on the carboxamide from a 3‐cyclohexyl propane resulted in a decrease in activity. For example, analogs **30** **–** **33** with an isopropyl, *tert*‐butyl, isobutyl, and cyclohexyl group all showed a decrease in antiplasmodial activity (EC_50_ 0.93–3.76 µM). Lastly, 3‐cyclopentyl propanamide **34** was also 2‐fold less active (EC_50_ 1.00 µM) compared to W482 (**1**) with a cyclohexyl group. Altogether, these results suggest that bulkier, more hydrophobic substitution on the carboxamide is important for maintaining antiparasitic activity.

**Table 5:** Activity of carboxamide analogs.

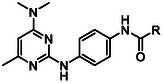			
Compound	*R*	Pf parasite EC_50_ (SD) [µM]a)	cLogPb)
W482 (**1**)	(CH_2_)_2_cyHex	0.49 (0.20)	4.6
**27**	2‐thiophene	1.44 (0.35)	3.7
**28**	2‐furan	3.90 (1.13)	3.0
**29**	2‐naphthyl	0.36 (0.09)	5.0
**30**	*i*Pr	3.76 (0.54)	3.0
**31**	*t*Bu	3.36 (0.96)	3.6
**32**	*i*Bu	1.63 (0.13)	3.5
**33**	cyHex	0.93 (0.23)	3.7
**34**	(CH_2_)_2_cyPen	1.00 (0.18)	4.3

a) EC_50_ data represent means and SDs for 3 or more experiments measuring LDH activity of *P. falciparum* 3D7 parasites following 72 h exposure to compounds.

b) cLogP was calculated using DataWarrior.^[^ ^34^ ^]^

The activity of 2‐naphthyl substituted derivative **29** pointed to investigating other hydrophobic substitutions on the aryl ring attached to the carboxamide. Derivatives **35** and **36** with 2‐chloro and 2‐bromo substitution had decreased antiparasitic activity (EC_50_ 2.12 and 1.85 µM), whereas compound **37** with 2‐trifluoromethyl substitution was 2‐fold less active (EC_50_ 0.85 µM) than W482 (**1**) (**Table** **6**). Analog **39** with 3‐bromo substitution was equipotent (EC_50_ 0.45 µM) to W482 (**1**) while variants **38** and **40** exhibited a 2‐fold lower antiplasmodial activity (EC_50_ 0.78 and 1.08 µM). Maintaining 3‐substitution, with additional substitution, was also examined. Compounds **41** and **42** with differing dimethyl substitution patterns exhibited similar potency (EC_50_ 0.59 and 0.66 µM) to W382 (**1**) but were not as potent as 2‐naphthyl **29** (EC_50_ 0.36 µM). Analogs **43** and **44** with 3,5‐dimethoxy and 3‐chloro, 6‐methoxy substitution, which were less lipophilic (cLogP 3.7 and 4.3), were equipotent (EC_50_ 0.64 and 0.49 µM) compared with W482 (**1**). Overall, some substituted aryl carboxamides showed similar activity to W482 (**1**), but none were as potent as the 2‐naphthyl substituted carboxamide analog **29**.

**Table 6:** Activity of substituted aryl carboxamide analogs.

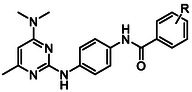			
Compound	R	Pf parasite EC_50_ (SD) [µM]a)	cLogPb)
**35**	2‐Cl	2.12 (0.30)	4.4
**36**	2‐Br	1.85 (0.41)	4.5
**37**	2‐CF_3_	0.85 (0.29)	4.6
**38**	3‐F	0.78 (0.45)	3.9
**39**	3‐Br	0.45 (0.11)	4.5
**40**	3‐Me	1.08 (0.17)	4.1
**41**	3,5‐diMe	0.59 (0.09)	4.5
**42**	3,4‐diMe	0.66 (0.16)	4.5
**43**	3,5‐diOMe	0.64 (0.18)	3.7
**44**	3‐Cl, 6‐OMe	0.49 (0.04)	4.3

a) EC_50_ data represent means and SDs for 3 or more experiments measuring LDH activity of *P. falciparum* 3D7 parasites following 72 h exposure to compounds.

b) cLogP was calculated using DataWarrior.^[^ ^34^ ^]^

Finally, a set of analogs containing various substituted aryl urea moieties replacing the carboxamide was assessed due to their structural similarity with the TCAMS hit, MMV020243 (**2**) (Figure 1). Analogs **45** and **46** with 2‐fluoro and 2‐chloro substitution had a significantly lower activity (EC_50_ 3.42 and > 10 µM) relative to W482 (**1**), while derivatives **47** and **48** with 2‐methyl and 2‐methoxy substitution were equipotent (EC_50_ 0.41 and 0.38 µM) (**Table** **7**). The compounds (**49**–**51**) with 3‐chloro, 3‐methyl, and 4‐fluoro substituents displayed a two‐fold increase in potency (EC_50_ 0.23–0.24 µM), whereas the 4‐chloro, 4‐methyl, and 4‐methoxy substituted derivatives **52**–**54** exhibited slightly decreased activity (EC_50_ 0.34–0.44 µM). Notably, the aryl urea analogs generally have reduced lipophilicity (cLogP 3.7–4.4) compared to aryl carboxamide analogs (cLogP 3.9–4.5), suggesting incorporation of the urea moiety may be beneficial for modulating the physicochemical properties. Moreover, halogen substitution in the 3‐ or 4‐position on the aryl urea moiety was beneficial for antiparasitic activity.

**Table 7:** Activity of urea‐based analogs.

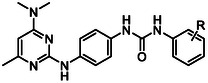			
Compound	R	Pf parasite EC_50_ (SD) [µM]a)	cLogPb)
MMV020243 (**2**)	H	1.15	3.0
**45**	2‐F	3.42 (0.35)	3.9
**46**	2‐Cl	>10	4.4
**47**	2‐Me	0.41 (0.09)	4.1
**48**	2‐OMe	0.38 (0.06)	3.7
**49**	3‐Cl	0.24 (0.04)	4.4
**50**	3‐Me	0.24 (0.02)	4.1
**51**	4‐F	0.23 (0.01)	3.9
**52**	4‐Cl	0.34 (0.01)	4.4
**53**	4‐Me	0.44 (0.10)	4.1
**54**	4‐OMe	0.40 (0.06)	3.7

a) EC_50_ data represent means and SDs for 3 or more experiments measuring LDH activity of *P. falciparum* 3D7 parasites following 72 h exposure to compounds.

b) cLogP was calculated using DataWarrior.^[^ ^34^ ^]^

Subsequently, two analogs were synthesized to assess whether a combination of modifications would improve antiparasitic activity. Analog **55** incorporated *N*‐piperazine substitution from compound **10** (Table 1) and the quinazoline core of **16** (Table 2), while analog **56** integrated the quinazoline core of **16** and the 4‐fluoroaryl urea moiety from compound **45** (Table 7). Both analogs **55** and **56** were found to exhibit enhanced activity (EC_50_ 0.23 and 0.18 µM) (**Table** **8**) relative to the progenitor compound **16** (EC_50_ 0.30 µM). Moreover, analog **56** had a lower cLogP (4.4) and may have contributed to enhanced aqueous solubility.

**Table 8:** Activity of carboxamide analogs.

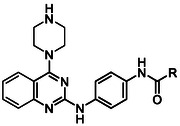			
Compound	R	Pf parasite EC_50_ (SD) [µM]a)	cLogPb)
**55**	(CH_2_)_2_cyHex	0.23 (0.03)	5.1
**56**	*N*‐(4‐F)‐aniline	0.18 (0.03)	4.4

a) EC_50_ data represent means and SDs for 3 or more experiments measuring LDH activity of *P. falciparum* 3D7 parasites following 72 h exposure to compounds.

b) cLogP was calculated using DataWarrior.^[^ ^34^ ^]^

**Figure 2:**
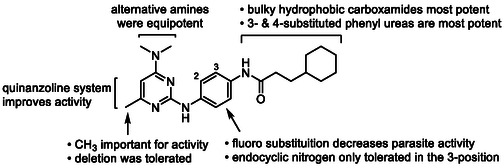
Summary of SAR.

In summary of the SAR (**Figure** **2**), the 4‐dimethylamine group could be replaced with a variety of substituted amine groups without loss of antiparasitic activity. The pyrimidine core and its endocyclic nitrogen configuration are important for antiparasitic activity, while the 6‐methyl group can be replaced by a 5,6‐arene system, but otherwise, the 6‐position is sensitive to modification. Alteration to the central ring led to a decrease in antiparasitic activity, although an endocyclic nitrogen in the 3‐position was tolerated. Bulkier and hydrophobic carboxamides or 3‐ and 4‐substituted phenyl ureas exhibit the most potent antiplasmodial activity. Hybrid analogs with a 4‐piperazine‐quinazoline moiety had marginally improved antiplasmodial activity. Overall, exploration and modification of the pyrimidine‐2,4‐diamine structure did not give rise to a major increase in potency, signifying that further improving the antiparasitic activity in the future will be challenging.

### 2.2 In Vitro ADME Analysis

The aqueous solubility and in vitro metabolic stability of selected analogs were assessed to determine if they had favorable parameters (**Table** **9**).These analogs were selected based on their potency and the modifications that were incorporated, which led to compounds **55** and **56**. The aqueous solubility at pH 7.4 of analogs **1**, **11**, **16**, **51,** and **55** was low (2.5–3.8 µM). For analogs **1**, **11**, and **16**, the low solubility was attributed to their high eLogD (4.6–5.0), while it was surprising that analogs **51** and **55** with lower eLogD (3.0 and 3.2) did not have increased aqueous solubility. Accordingly, analog **56** had slightly improved solubility (9.6 µM) likely due to the inclusion of an aliphatic piperazine ring and urea functionality contributing to the lower eLogD (2.2). All analogs, however, exhibited high albumin protein binding (96.6–99.9% bound). The high lipophilicity of analogs **1**, **11**, and **16** was not favorable for metabolic stability. This was evident with analogs **1**, **11**, **16**, **55,** and **56** showing high clearance in human liver microsomes (CL_int_ > 117 µL/min/mg), except for derivative **51** that exhibited significantly lower clearance (CL_int_ 26 µL/min/mg). Analogs **1**, **11**, and **16** also showed high clearance in rat hepatocytes (>92 µL/min/10^6^ cells), while clearance observed with analogs **51**, **55**, and **56** was significantly lower (21–32 µL/min/10^6^ cells). Despite **51** having low aqueous solubility, it exhibited the best balance of metabolic stability and antiparasitic activity (EC_50_ 0.30 µM). Altogether, the in vitro ADME analysis revealed that further improvement of the physicochemical parameters while improving antiparasitic activity will be challenging.

**Table 9:** In vitro ADME values for selected compounds.

Compound	Aqueous solubility pH 7.4 [µM]a)	Human liver microsomes CL_int_ [µL/min/mg]	Rat hepatocytes CL_int_ [µL/min/10^6^ cells]	eLogDb)	Albumax binding % bound
W482 (**1**)	3.8	174	>92	4.9	99.1
**11**	2.6	327	>92	5.0	99.9
**16**	2.5	166	>92	4.6	96.6
**51**	3.7	26	32	3.0	98.4
**55**	2.5	117	27	3.2	99.8
**56**	9.6	266	21	2.2	99.9

a) Kinetic in PBS.

b) Shake‐flask method.

### 2.3 Synthesis

**Scheme 1:**
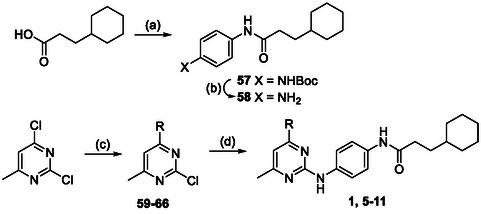
Synthesis of 4‐substituted amine analogs. *Reagents and conditions:* a) (i) oxalyl chloride, DCM, cat. DMF, 20 °C, 1 h; (ii) *tert*‐butyl (4‐aminophenyl)carbamate, Et_3_N, DCM, 20 °C, 2 h; b) TFA, DCM, 20 °C, 2 h; c) aliphatic amine, DIPEA, DMF, 20 °C, 18 h; d) **58**, *tert*‐butanol, reflux, 18 h. *R* = aliphatic amine substituents shown in Table 1.

**Scheme 2:**
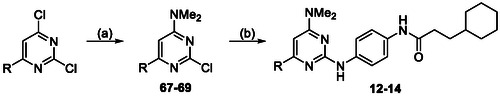
Synthesis of 6‐substituted analogs. *Reagents and conditions:* a) dimethylamine, DIPEA, DMF, 20 °C, 18 h; b) **58**, *tert*‐butanol, reflux, 18 h. *R* = substituents shown in Table 2.

**Scheme 3:**
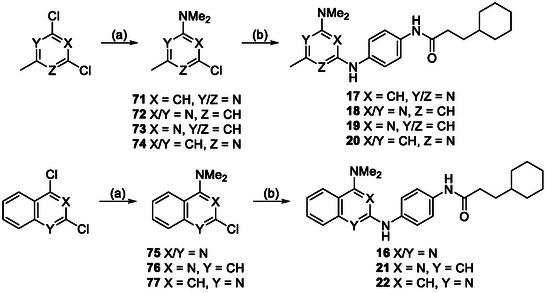
Synthesis of analogs with alternate heterocycles. *Reagents and conditions:* a) dimethylamine, DIPEA, DMF, 20 °C, 18 h; b) **58**, *tert*‐butanol, reflux, 18 h for **16–18** or **58**, G3‐Pd‐xantphos, Cs_2_CO_3_, 1,4‐dioxane, µW, 120 ºC, 1 h for **19–22**. Analog variations are shown in Table 3.

**Scheme 4:**
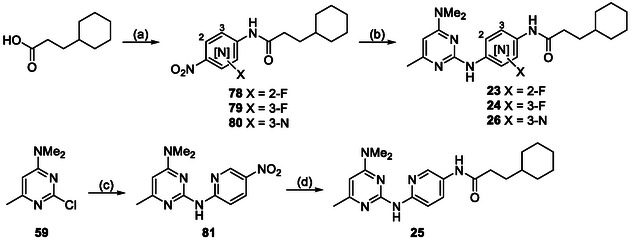
Synthesis of analogs with central ring modifications. *Reagents and conditions:* a) 4‐nitro‐aniline, T3P, DIPEA, DCE, µW, 130 ºC, 1 h; b) (i) Pd/C, H_2_, methanol, 20 °C, 1 h; (ii) **59**, *tert*‐butanol, reflux, 18 h. c) 5‐nitropyridin‐2‐amine, *tert*‐butanol, reflux, 18 h; d) (i) Pd/C, H_2_, methanol, 20 °C, 1 h; (ii) 3‐cyclohexylpropanoic acid, T3P, DIPEA, DCE, µW, 130 ºC, 1 h. Analog variations are shown in Table 4.

**Scheme 5:**
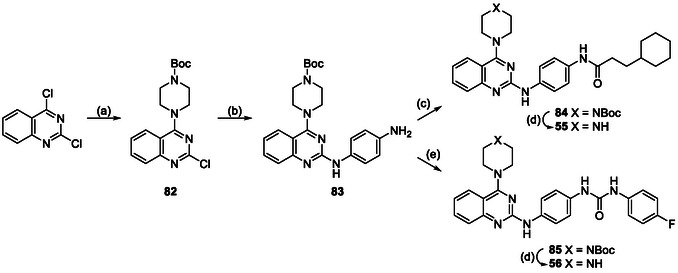
Synthesis of analogs with carboxamide variations. *Reagents and conditions:* a) Boc‐piperazine, DIPEA, DMF, 20 °C, 18 h; b) (i) 4‐nitroaniline, G3‐Pd‐xantphos, Cs_2_CO_3_, 1,4‐dioxane, µW, 120 ºC, 1 h; (ii) Pd/C, H_2_, methanol, 20 °C, 1 h; c) (i) oxalyl chloride, cat. DMF, DCM, 20 °C, 1 h; (ii) Et_3_N, DCM, 20 °C, 2 h; d) 4N HCl, dioxane, 20 °C, 18 h; e) (i) 4‐nitrophenyl chloroformate, THF, 20 °C, 30 min; (ii) 4‐fluoroaniline, Et_3_N, THF, 20 °C, 20 h.

The pyrimidine‐2,4‐diamine derivatives **1**, **5**–**26**, **55,** and **56** in this study were synthesized using the following synthetic routes (**Scheme** **1**–**5**). Compounds **4** and **27**–**54** were purchased commercially, and the integrity and purity were confirmed by liquid chromatography mass spectrometers (LCMS). The synthesis of analogs **1, 5–11** with varying substitution in the 4‐position (Table 1) began with the preparation of the aniline fragment (Scheme 1). This was constructed by reacting 3‐cyclohexylpropanoic acid with oxalyl chloride to produce an acid chloride, which was on reacted with *tert*‐butyl (4‐aminophenyl)carbamate to give the carboxamide **57**. Boc‐deprotection of **57** yielded the *N*‐substituted anilide **58**. For the synthesis of the pyrimidine component, a nucleophilic aromatic substitution reaction between 2,4‐dichloro‐6‐methylpyrimidine and an amine under mild conditions yielded 4‐substituted pyrimidine intermediates **59**–**66**. These intermediates were then used in a nucleophilic aromatic substitution reaction with anilide **58** under reflux in *tert*‐butanol to yield the final products **1, 5**–**11**.

The synthesis of analogs **12**–**14** with modifications to the 6‐position (Table 2) began with a nucleophilic aromatic substitution reaction between 2,4‐dichloro‐6‐substituted pyrimidines and dimethylamine in DMF at 20 °C to yield the 4‐substituted pyrimidine intermediates **67**–**69** (Scheme 2). These intermediates were reacted with the anilide **58** at an elevated temperature to produce 6‐substituted pyrimidine analogs **12**–**14**. Analogs **16**–**22** with varying endocyclic nitrogen makeup (Table 3) were synthesized following a similar pathway. The synthesis started by reacting either a dichloro‐substituted 6‐membered heterocycle or a dichloro‐substituted biaryl heterocycle system with dimethyl amine in DMF at 20°C to yield the dimethyl amino substituted heterocycle intermediates **71**–**77** (Scheme 3). These intermediates were then reacted with the anilide **58** in *tert‐*butanol at reflux (for activated systems) or using Buchwald conditions (for deactivated systems) to give the heterocyclic analogs **17**–**22**.

The synthesis of analogs **23**, **24,** and **26** with fluoro substitution or a 3‐endocyclic nitrogen in the central aryl ring (Table 4) started by reacting 3‐cyclohexylpropanoic acid with the appropriate 4‐nitro aniline using T3P under microwave irradiation to produce the amide intermediates **78**, **79,** and **80** (Scheme 4). The nitro group on the intermediates was then reduced under hydrogenation conditions, and the subsequent amine products, on‐reacted with the 4‐dimethylamino‐6‐methyl pyrimidine **59** at elevated temperature in *tert*‐butanol to give the derivatives **23**, **24,** and **26**. For the synthesis of analog **25** (Table 4), 4‐dimethylamino‐6‐methyl pyrimidine **59** was reacted with 5‐nitropyridine‐2‐amine under reflux in *tert*‐butanol to afford the 2‐substituted pyrimidine intermediate **81** (Scheme 4). Subsequently, the nitro group was reduced using catalytic palladium on charcoal under an atmosphere of hydrogen in methanol to give an aniline intermediate which was on‐reacted with 3‐cyclohexylpropanoic acid using T3P under microwave irradiation to give analog **25**.

The synthesis of analogs **55** and **56** with a combination of modifications (Table 8), started with reacting the 2,4‐dichloroquinazoline Boc‐*N*‐piperazine in the presence of Hunig's base in DMF at 20°C to yield the 4‐substituted quinazoline **82** (Scheme 5). **82** was then reacted with 4‐nitro‐aniline under Buchwald conditions using catalytic G3‐Pd‐xantphos and cesium carbonate in dioxane under microwave irradiation to yield the 2‐substituted quinazoline intermediate **83**. **83** was then reacted with the in situ generated acid chloride of 3‐cyclohexylpropanoic acid in the presence of triethylamine to give the carboxamide **84**. Intermediate **83** was also reacted with 4‐nitrophenyl chloroformate in THF to give the 4‐nitrophenyl carbamate functionality, which was on‐reacted with 4‐fluoroaniline in the presence of triethylamine to give the urea **85**. Both **84** and **85** were subsequently subject to acidic conditions to deprotect the Boc group to afford the derivatives **55** and **56**.

### 2.4 Resistance Selection and Whole Genome Sequencing

**Figure 3:**
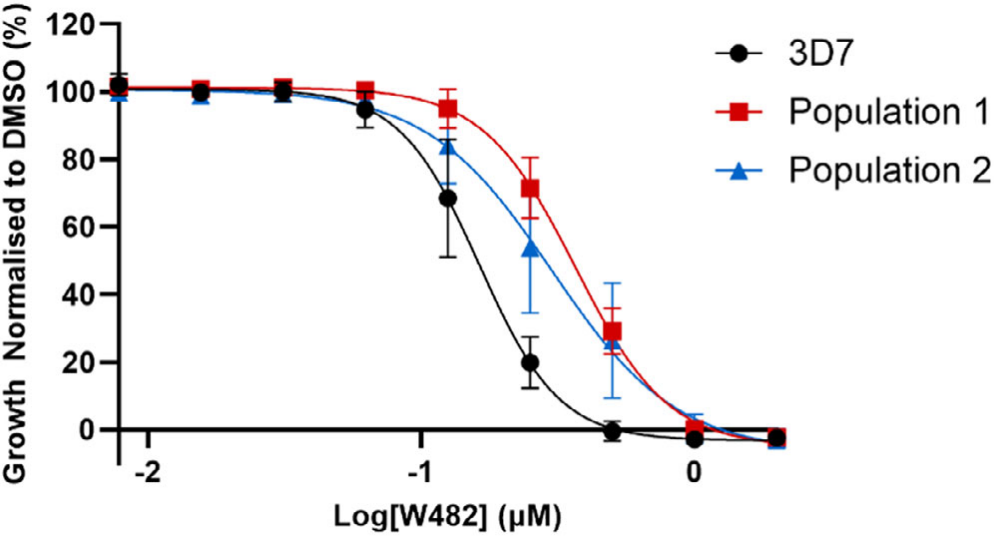
The activity of W482 (**1**) against W482‐selected populations and their 3D7 parental. The data are mean and SD from 3 independent 72 h *Pf* LDH assays.

To identify the mechanism by which the pyrimidine‐2,4‐diamine class kills the *P. falciparum* parasite, a forward genetic study was performed on the hit compound W482 (**1**). We selected W482 (**1**) as the representative compound from this class, as the forward genetics and phenotype assays were performed in parallel to the medicinal chemistry described in the prior section. The forward genetics study involved treating three independent *P. falciparum* 3D7 parasite populations with W482 (**1**), with the dose increased incrementally from 2 × EC_50_ (0.3 µM) to 10 × EC_50_ (1.5 µM). After this treatment, two of the three populations recovered and were found to have 2‐fold decreased activity against W482 (**1**) compared to the parental 3D7 parasites (EC_50_ 0.197 µM for 3D7, 0.396 µM for population #1, and 0.407 µM for population #2) (**Figure** **3**). Genomic DNA was then prepared from these two resistant populations and the parental 3D7 strain and whole genome sequenced.

Whole genome sequencing revealed single‐nucleotide polymorphisms (SNPs) and a copy number variation (CNV) in both W482‐resistant populations #1 and #2. Out of the 9 SNPs identified in the coding region, only two were nonsynonymous (Table S3, Supporting Information). The first of these (PF3D7_1036400, LSA1) is in a region with little to no coverage and has low quality. The latter (PF3D7_1303800) is in an unnamed conserved protein with an unknown function and is likely to be low impact, as both Ile and Met are neutral amino acids with medium‐sized hydrophobic side chains.

Copy number analysis uncovered duplication events in chromosome 3, which were different but overlapping in the two pairs of samples (Figure S2 and S3; Table S1, Supporting Information). In population #1, a 2‐fold amplification of chromosome 3 (between positions 808,662–887,962) was observed, while population #2 replicates each displayed a 1.5‐ and 2‐fold amplification in chromosome 3 (723,273–836,978 and 743,361–876,137). Notably, reviewing the genes present in the amplification region (Table S2, Supporting Information) exposes ABCI3 (PF3D7_0319700), a putative transmembrane spanning ABC transporter I family member 3 protein.^[^ ^26^ ^]^ In *P. falciparum,* the ABCI3 transporter has been proposed to mediate resistance to several compound classes by transporting them across membranes away from the site of the molecular target.^[^ ^19^ ^,^ ^26^ ^]^ Further research on the role of ABCI3 could aid the understanding of the compound‐transporter relationship.

### 2.5 Evaluation against Parasites with Reduced Expression of ABCI3

**Figure 4:**
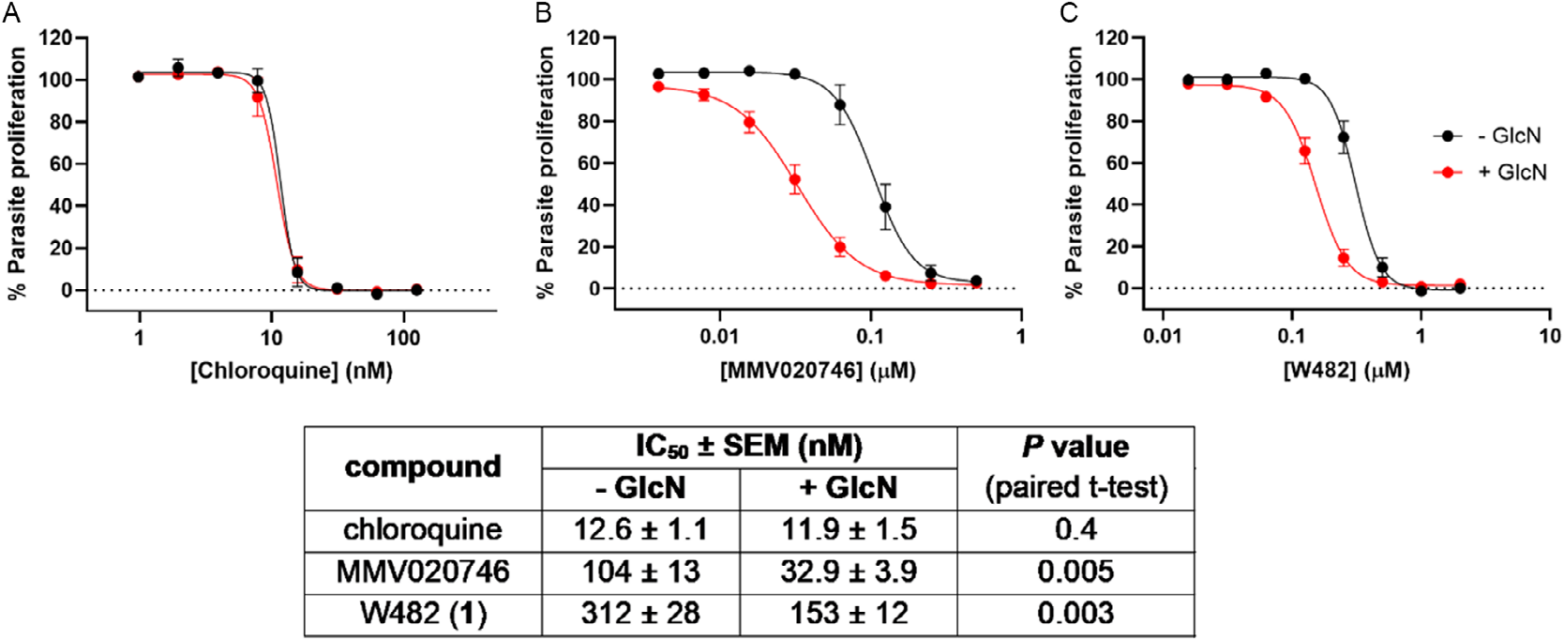
Sensitivity of PfABCI3‐HAreg parasites to growth inhibition by chloroquine (a), MMV020746 (b), and W482 (**1**) (c). Data for parasites in which *pfabci3‐ha* mRNA was reduced (+ GlcN) are shown in red. GlcN (5 mM) was added to the parasites 48 h prior to the assay and maintained throughout the 72 h assay. The data for parasites expressing a normal level of PfABCI3‐HA (‐GlcN) are shown in black. The data in all panels are from four independent experiments performed on different days. The Table shows IC_50_ values (mean ± SEM, *n* = 4) derived from the experiments shown in a–c. Parasites that were treated with or without GlcN (5 mM) were tested concurrently in each assay.

To support the findings of this forward genetics study that suggested that the PfABCI3 transporter is associated with W482‐resistance, we tested the antiplasmodial activity of W482 (**1**) with a parasite line in which the endogenous *pfabci3* gene is fused at its 3′ end to an HA tag (3 × HA) and *glmS* ribozyme (“PfABCI3‐HAreg”; described previously^[^ ^19^ ^]^). Glucosamine (GlcN) (5 mM) was used to reduce the level of *pfabci3‐ha*. Chloroquine and MMV020746 were used as negative and positive controls, respectively. Consistent with previous results,^[^ ^19^ ^,^ ^26^ ^]^ chloroquine was equally potent against control parasites expressing a normal level of PfABCI3‐HA (‐GlcN) and parasites in which *pfabci3‐ha* mRNA was reduced (+ GlcN) (**Figure** **4**a). MMV020746 was more potent against +GlcN parasites than against ‐GlcN parasites (Figure 4b), with a 3.2 ± 0.2 fold lower IC_50_ value for the former (mean ± SEM, *n* = 4), consistent with previous findings for this compound obtained with a parasite line in which PfABCI3 expression levels were controlled using the TetR‐DOZI system.^[^ ^26^ ^]^ W482 (**1**) was found to be more potent against +GlcN parasites than against ‐GlcN parasites (Figure 4c), with the +GlcN parasites having a 2.0 ± 0.1 fold lower IC_50_ value (mean ± SEM, *n* = 4). This supports the whole genome sequencing results, suggesting that the compound either targets PfABCI3 or succumbs to a PfABCI3‐mediated resistance mechanism.

### 2.6 Asexual Stage and Rate of Arrest

**Figure 5:**
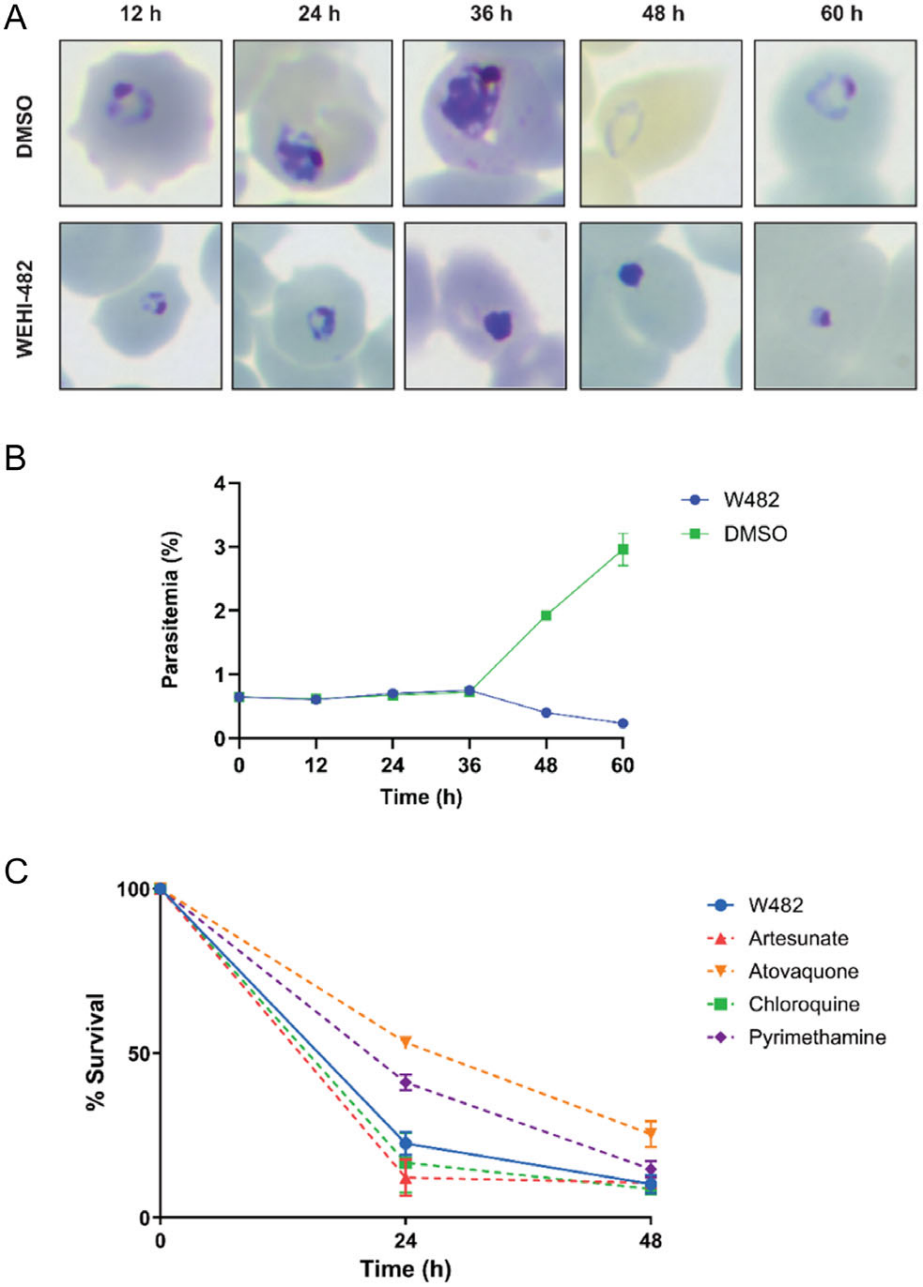
a) Stage of arrest assay showing that W482 (**1**) blocks ring‐trophozoite transition in the asexual stage of *P. falciparum*. Ring‐stage parasites were exposed to 10 × EC_50_ of W482 (**1**) (1.4 µM) for 60 h, with samples taken every 12 h for Giemsa‐stained blood smears (a) and b) parasitemia quantification via SYBR Green staining and flow cytometry Data points represent the average of three technical replicates for parasitemia. Replicates of Giemsa‐stained blood smears can be found in Figure S4 (Supporting Information). c) Parasite reduction ratio assay conducted at 10 × EC_50_ of W482 (**1**). Data represent means and SD of *P. falciparum* 3D7 parasite survival measuring 3 independent replicates by two‐color flow cytometry using CFDA‐SE and Hoescht 33,342 staining.

The asexual stage of arrest and parasite reduction ratio (PRR) assays were undertaken on W482 (**1**) to assess the speed‐of‐asexual kill and the alignment of the pyrimidine‐2,4‐diamine class with the Target Candidate Profiles (TCPs).^[^ ^27^ ^]^ To determine the asexual stage of arrest, *P. falciparum* 3D7 parasites were treated with W482 (**1**) at 10 × EC_50_ (1.4 µM) for 60 h. Parasite samples were collected from the culture at 12, 24, 36, 48, and 60 h, and microscopy was performed on Giemsa‐stained thin blood‐smeared samples. These samples were also quantified with flow cytometry using SYBR Green. It was demonstrated that W482 (**1**) arrested asexual parasites at the ring to trophozoite transition stage (**Figure** **5**a and S4, Supporting Information). Quantification of parasite growth revealed that the percentage of parasitemia declines after 36 h (Figure 5b).

To determine the asexual speed‐of‐kill profile of W482 (**1**), synchronized *P. falciparum* 3D7 parasites were treated with 10 × EC_50_ of W482 (**1**) for 48 h. Samples of parasites were collected at 24‐ and 48‐hour timepoints, whereby the compound was washed out and fresh erythrocytes pre‐labeled with intracellular dye were added. Samples were then incubated for 18 days, and then the number of viable parasites was quantified by flow cytometry (Figure 5c). Pyrimethamine was used as an internal control, demonstrating a moderate killing rate as reported previously.^[^ ^28^ ^]^ Hit compound W482 (**1**) displayed a fast killing profile similar to that of chloroquine or artemisinin.

## 3 Conclusion

The series of pyrimidine‐2,4‐diamine derivatives was designed and evaluated for their activity against *P. falciparum* 3D7 parasites. The SAR was explored, and demonstrated that while the pyrimidine core and its endocyclic nitrogens are important for antiparasitic activity, a variety of substituted amines are tolerated in the 4‐position. Central ring modifications led to a decrease in antiplasmodial activity, although an endocyclic nitrogen in the 3‐position is tolerated. Bulky and hydrophobic carboxamides or substituted phenyl ureas displayed the most potent antiplasmodial activity of this class. Pyrimidine‐2,4‐diamine analogs generally displayed low metabolic stability and low aqueous solubility, which may be connected to the high eLogD, except for analog **51,** which had a reasonable balance of metabolic stability and antiparasitic activity. Though the preliminary SAR of this scaffold was established, future improvements in antiparasitic activity, metabolic stability, and solubility will be challenging to achieve.

Forward genetics studies showed an amplification of the *abci3* gene, suggesting W482 (**1**) either targets PfABCI3 or is vulnerable to a PfABCI3‐mediated resistance mechanism. Further evaluation confirmed this finding, with W482 (**1**) found to be more potent against parasites with reduced expression of PfABCI3. Notably, previous metabolomic profiling on the MMV Malaria Box analog MMV396669 **3** (Figure 1) demonstrated a peptide signature consistent with hemoglobin catabolism inhibition.^[^ ^29^ ^]^ Further studies are required to understand whether inhibition of hemoglobin catabolism is the mechanism of action of the pyrimidine‐2,4‐diamine class. Moreover, determine whether ABCI3 is indeed a mechanism of resistance that transports the compound out of the compartment that houses the molecular target of the pyrimidine‐2,4‐diamine series.

While the pyrimidine‐2,4‐diamine structural class has fast‐to‐moderate speed of kill and modest activity against asexual stage parasites, data on the MMV Malaria Box analogs, MMV020243 (**2**) and MMV396669 (**3**) (Figure 1), show these compounds exhibit weak activity against early stage (I–III) gametocytes (67% and 79% inhibition at 2.5 µM) and no activity against late stage (IV‐V) gametocytes (2% and 21% inhibition at 5 µM).^[^ ^25^ ^]^ It was also found that these compounds have no activity against *P. berghei* liver stage development (2% and 3% inhibition at 50 µM).^[^ ^25^ ^]^ Though the activity of the analogs generated here against the transmission and liver stages needs verification, it would be unlikely that the pyrimidine‐2,4‐diamine class has potential as a seasonal transmission‐blocking agent in malaria‐endemic areas or as a causal chemopreventative agent.^[^ ^27^ ^]^ Overall, this research established that the pyrimidine‐2,4‐diamine class represents a low‐priority scaffold for further antimalarial development.

## 4 Experimental Section

4.1

4.1.1

##### Chemistry Experimental: General Chemistry Methods

NMR spectra were recorded on a Bruker Ascend 300. Spectra were processed using MestReNova software. Chemical shifts (*δ*) are reported in parts per million (ppm) and referenced to the corresponding solvent signal of either Chloroform*d* (CDCl_3_) or DMSO*d*_6;_ these solvents contain H_2_O. Coupling constants (*J*) are recorded in Hertz (Hz) and multiplicities are described by singlet (s), broad singlet (bs), doublet (d), triplet (t), quartet (q), doublet of doublets (dd), doublet of triplets (dt), doublet of doublet of doublets (ddd) and multiplet (m). Chromatography was performed with silica gel 60 (particle size 0.040–0.063 µm) using an automated CombiFlash Rf or Biotage Selekt Purification System. LCMS were recorded using Agilent G6120B MSD, using 1260 Infinity G1312B binary pump with 1260 detector Infinity G4212B DAD, the LC conditions as it follows, column; Poroshell 120 EC‐C18, 2.1 × 30 mm 2.7 micron, injection volume: 1 µL; flow rate 1.0 mL min^–^^1^; gradient 5–100% of B over 3.8 min, (Solvent A: water, 0.1% formic acid; solvent B: ACN, 0.1% formic acid). MS conditions were as follows, ion source: single‐quadrupole, ion mode: ES positive unless otherwise specified, source temperature: 150 °C, desolvation temperature: 350 °C, detection: ion counting, Capillary (KV)−3.00, Cone(V): 30, Extractor (V): 3, RF Lens (V): 0.1, Scan Range: 100–1000 Amu, Scan Time: 0.5 s, Acquisition time: 4.1 min, Gas flow: desolvation L/hr‐650, Cone L/hr‐100. Alternatively, Agilent G6120B MSD, using 1260 Infinity II G7129B binary pump with 1290 detector Infinity G71172B DAD, the LC conditions as it follows, column Luna, Omega 3.0 μM PS C18 100 A, 50 x 2.1 mm; injection volume: 1 μL; flow rate 0.6 mL min^–1^; gradient 5–100% of B over 3.8 min, (solvent A: water, 0.1% formic acid; solvent B: ACN, 0.1% formic acid). MS conditions were as follows, ion source: single‐quadrupole, ion mode: ES positive unless otherwise specified, source temperature: 150 °C, desolvation temperature: 350 °C, detection: ion counting, Capillary (KV)−3.00, Cone(V): 30, Extractor (V): 3, RF Lens (V): 0.1, Scan Range: 100–1000 Amu, Scan Time: 0.5 s, Acquisition time: 4.1 min, Gas flow: desolvation 650 L h^–^^1^, Cone 100 L h^–1^. Unless otherwise noted, all compounds were found to be >95% pure by this method.

Compounds **4** and **27**–**54** were purchased commercially. The integrity and purity of these compounds were confirmed to be >95% by LCMS.

##### General Procedure A: 3‐Cyclohexyl‐*N*‐(4‐((4‐(dimethylamino)‐6‐methylpyrimidin‐2‐yl)amino)phenyl)propenamide (1) (W482)

Aniline **58** (34 mg, 0.14 mmol, 1 eq.) and arylchloride intermediate **59** (24 mg, 0.14 mmol, 1 eq.) were dissolved in *tert*‐butanol (3 mL) and heated to 115 °C overnight. After complete conversion was observed in LCMS, the mixture was concentrated to dryness, and the crude residue was purified using flash column chromatography (0–5% MeOH/DCM) to afford **1** as an off‐white powder (12 mg, 23%). ^1^H NMR (300 MHz, DMSO) *δ* 9.67 (s, 1H), 8.92 (s, 1H), 7.63 (d, *J* = 8.5 Hz, 2H), 7.42 (d, *J* = 8.6 Hz, 2H), 5.99 (s, 1H), 3.04 (s, 6H), 2.28 (t, *J* = 8.2 Hz, 2H), 2.18 (s, 3H), 1.76–1.58 (m, 5H), 1.52–1.43 (m, 2H), 1.27–1.07 (m, 4H), 0.95–0.83 (m, 2H). ^13^C NMR (75 MHz, DMSO) *δ* 170.9, 162.9, 132.7, 119.4, 118.8, 92.8, 79.4, 78.5, 36.8, 36.8, 33.9, 32.7, 32.6, 26.1, 25.8, 23.4, 2.8. LCMS *m/z* 382.3 [M + H]^+^.

##### 3‐Cyclohexyl‐*N*‐(4‐((4‐(ethyl(methyl)amino)‐6‐methylpyrimidin‐2‐yl)amino)phenyl)propenamide (5)

General Procedure A was followed using **58** (30 mg, 0.12 mmol) and **60** (23 mg, 0.12 mmol) to afford **5** as a white powder (36 mg, 73%). ^1^H NMR (300 MHz, DMSO) *δ* 9.69 (s, 1H), 8.84 (s, 1H), 7.63 (d, *J* = 8.9 Hz, 2H), 7.41 (d, *J* = 8.9 Hz, 2H), 5.94 (s, 1H), 3.54–3.51 (m, 2H), 2.98 (s, 3H), 2.26 (t, *J* = 7.7 Hz, 2H), 2.16 (s, 3H), 1.75–1.57 (m, 5H), 1.47 (q, *J* = 7.2 Hz, 2H), 1.34–1.12 (m, 4H), 1.08 (t, *J* = 7.0 Hz, 3H), 0.94–0.81 (m, 2H). ^13^C NMR (75 MHz, DMSO) *δ* 171.1, 164.8, 162.2, 159.1, 136.9, 132.6, 119.5, 118.6, 92.7, 43.4, 36.9, 34.6, 34.0, 32.8, 32.7, 26.2, 25.8, 23.7, 11.9. LCMS *m/z* 396.4 [M + H]^+^.

##### *N*‐(4‐((4‐(Benzyl(methyl)amino)‐6‐methylpyrimidin‐2‐yl)amino)phenyl)‐3‐cyclohexylpropanamide (6)

General Procedure A was followed using **58** (20 mg, 0.08 mmol) and **61** (20 mg, 0.08 mmol) to afford **6** as an off‐white powder (17 mg, 46%). ^1^H NMR (300 MHz, DMSO) *δ* 9.64 (s, 1H), 8.83 (s, 1H), 7.54 (d, *J* = 8.6 Hz, 2H), 7.40–7.26 (m, 4H), 7.26–7.16 (m, 3H), 6.02 (s, 1H), 4.78 (s, 2H), 3.02 (s, 3H), 2.24 (t, *J* = 7.7 Hz, 2H), 2.17 (s, 3H), 1.71–1.60 (m, 4H), 1.46 (q, *J* = 7.3 Hz, 2H), 1.27–1.07 (m, 5H), 0.93–0.80 (m, 2H). ^13^C NMR (75 MHz, DMSO) *δ* 171.2, 165.6, 162.9, 159.3, 138.4, 136.8, 132.6, 128.6, 127.0, 126.9, 119.5, 118.7, 92.8, 51.8, 36.9, 35.7, 34.0, 32.8, 32.7, 26.3, 25.9, 23.9. LCMS *m/z* 458.4 [M + H]^+^.

##### 3‐Cyclohexyl‐*N*‐(4‐((4‐methyl‐6‐(pyrrolidin‐1‐yl)pyrimidin‐2‐yl)amino)phenyl)propanamide (7)

General Procedure A was followed using **58** (35 mg, 0.14 mmol) and **62** (35 mg, 0.14 mmol) to afford **7** as a white powder (32 mg, 55%). ^1^H NMR (300 MHz, DMSO) *δ* 9.67 (s, 1H), 8.99 (s, 1H), 7.67 (d, *J* = 8.6 Hz, 2H), 7.43 (d, *J* = 8.6 Hz, 2H), 5.82 (s, 1H), 3.41 (bs, 4H), 2.27 (t, *J* = 7.7 Hz, 2H), 2.17 (s, 3H), 1.93 (d, *J* = 6.2 Hz, 4H), 1.77–1.58 (m, 5H), 1.48 (q, *J* = 7.3 Hz, 2H), 1.29–1.11 (m, 4H), 0.96–0.79 (m, 2H). ^13^C NMR (75 MHz, DMSO) *δ* 170.9, 162.9, 160.5, 158.2, 136.4, 132.7, 119.4, 118.7, 93.7, 46.1, 36.8, 33.9, 32.7, 32.6, 25.9, 25.7, 24.7, 22.9. LCMS *m/z* 408.2 [M + H]^+^.

##### 3‐Cyclohexyl‐*N*‐(4‐((4‐methyl‐6‐(piperidin‐1‐yl)pyrimidin‐2‐yl)amino)phenyl)propenamide (8)

General Procedure A was followed using **58** (30 mg, 0.14 mmol) and **63** (28 mg, 0.14 mmol) to afford **8** as a white powder (56 mg, 93%). ^1^H NMR (300 MHz, DMSO) *δ* 9.69 (s, 1H), 8.93 (s, 1H), 7.58 (d, *J* = 8.7 Hz, 2H), 7.43 (d, *J* = 8.9 Hz, 2H), 6.14 (s, 1H), 3.58 (t, *J* = 5.2 Hz, 4H), 2.27 (t, *J* = 7.7 Hz, 2H), 2.17 (s, 3H), 1.74–1.60 (m, 7H), 1.48 (dd, *J* = 15.0, 7.1 Hz, 6H), 1.24–1.12 (m, 4H), 0.94–0.82 (m, 2H). ^13^C NMR (75 MHz, DMSO) *δ* 170.9, 162.2, 158.8, 136.4, 132.8, 119.4, 118.9, 115.0, 93.1, 44.7, 36.8, 33.9, 32.7, 32.6, 26.1, 25.8, 25.2, 24.3, 23.4. LCMS *m/z* 422.4 [M + H]^+^.

##### 3‐Cyclohexyl‐*N*‐(4‐((4‐methyl‐6‐morpholinopyrimidin‐2‐yl)amino)phenyl)propenamide (9)

General Procedure A was followed using **58** (33 mg, 0.13 mmol) and **64** (29 mg, 0.13 mmol) to afford **9** as an off‐white powder (31 mg, 55%). ^1^H NMR (300 MHz, DMSO) *δ* 9.68 (s, 1H), 8.96 (s, 1H), 7.58 (d, *J* = 9.0 Hz, 2H), 7.42 (d, *J* = 8.7 Hz, 2H), 6.12 (s, 1H), 3.66 (t, *J* = 4.7 Hz, 4H), 3.53 (t, *J* = 4.8 Hz, 4H), 2.26 (t, *J* = 7.7 Hz, 2H), 2.18 (s, 3H), 1.75–1.59 (m, 5H), 1.47 (q, *J* = 7.3 Hz, 2H), 1.25–1.11 (m, 4H), 0.96–0.83 (m, 2H). ^13^C NMR (75 MHz, DMSO) *δ* 170.9, 165.8, 163.0, 159.2, 136.6, 132.7, 119.4, 118.8, 93.2, 65.9, 44.1, 36.8, 33.9, 32.7, 32.6, 26.1, 25.8, 23.8. LCMS *m/z* 424.3 [M + H]^+^.

##### 3‐Cyclohexyl‐*N*‐(4‐((4‐methyl‐6‐(piperazin‐1‐yl)pyrimidin‐2‐yl)amino)phenyl)propenamide (10)

General Procedure A was followed using **58** (40 mg, 0.16 mmol) and **65** (51 mg, 0.16 mmol) to form the Boc‐protected intermediate as a white powder (52 mg, 61%). This intermediate was dissolved in DCM (4 mL), and trifluoroacetic acid (385 µL, 0.38 mmol) was slowly added, and the mixture was left to react at room temperature. Complete conversion was observed after 2 h. The volatiles were removed under a stream of N_2,_ and the desired product was triturated from the crude residue using diethyl ether and filtered to afford **10** as an off‐white solid (23 mg, 33% yield over the two steps). ^1^H NMR (300 MHz, DMSO) *δ* 10.57 (s, 1H), 9.92 (s, 1H), 9.13 (s, 1H), 7.62 (d, *J* = 8.9 Hz, 2H), 7.43 (d, *J* = 8.6 Hz, 2H), 6.59 (s, 1H), 4.01–3.91 (m, 4H), 3.28–3.19 (m, 4H), 2.36–2.26 (m, 5H), 1.74–1.58 (m, 5H), 1.49 (q, *J* = 7.3 Hz, 2H), 1.26–1.10 (m, 4H), 0.95–0.83 (m, 2H). ^13^C NMR (75 MHz, DMSO) *δ* 171.4, 161.8, 159.1, 158.7, 136.0, 132.1, 121.9, 119.5, 94.5, 42.2, 41.4, 36.8, 33.9, 32.6, 32.6, 26.1, 25.8, 19.1. LCMS *m/z* 423.4 [M + H]^+^.

##### 3‐Cyclohexyl‐*N*‐(4‐((4‐methyl‐6‐(4‐methylpiperazin‐1‐yl)pyrimidin‐2‐yl)amino)phenyl)propenamide (11)

General Procedure A was followed using **58** (30 mg, 0.12 mmol) and **66** (28 mg, 0.12 mmol) to afford **11** as a white powder (26 mg, 49%). ^1^H NMR (300 MHz, DMSO) *δ* 9.69 (s, 1H), 8.86 (s, 1H), 7.58 (d, *J* = 8.9 Hz, 2H), 7.41 (d, *J* = 8.8 Hz, 2H), 6.11 (s, 1H), 3.57–3.53 (m, 4H), 2.39–2.31 (m, 4H), 2.26 (t, *J* = 7.7 Hz, 2H), 2.20 (s, 3H), 2.16 (s, 3H), 1.75–1.57 (m, 5H), 1.47 (q, *J* = 7.3 Hz, 2H), 1.27–1.12 (m, 4H), 0.87 (q, *J* = 10.6 Hz, 2H). ^13^C NMR (75 MHz, DMSO) *δ* 171.1, 165.8, 162.8, 159.4, 136.7, 132.6, 119.6, 118.8, 93.3, 54.3, 45.8, 43.6, 36.9, 34.0, 32.8, 32.7, 26.2, 25.8, 23.9. LCMS *m/z* 437.4 [M + H]^+^.

##### 3‐Cyclohexyl‐*N*‐(4‐((4‐(dimethylamino)pyrimidin‐2‐yl)amino)phenyl)propenamide (12)

General Procedure A was followed using **58** (35 mg, 0.14 mmol) and **67** (22 mg, 0.14 mmol) to afford **12** as a white powder (25 mg, 48%). ^1^H NMR (300 MHz, DMSO) *δ* 9.69 (s, 1H), 8.87 (s, 1H), 7.89 (d, *J* = 6.0 Hz, 1H), 7.63 (d, *J* = 8.8 Hz, 2H), 7.42 (d, *J* = 8.8 Hz, 2H), 6.07 (d, *J* = 6.0 Hz, 1H), 3.04 (s, 6H), 2.26 (t, *J* = 7.7 Hz, 2H), 1.75–1.56 (m, 5H), 1.47 (q, *J* = 7.3 Hz, 2H), 1.26–1.09 (m, 4H), 0.95–0.80 (m, 2H). ^13^C NMR (75 MHz, DMSO) *δ* 171.0, 162.3, 159.4, 156.0, 136.7, 132.6, 119.5, 118.8, 94.5, 36.9, 36.7, 33.9, 32.8, 32.7, 26.2, 25.8. LCMS *m/z* 368.2 [M + H]^+^.

##### 3‐Cyclohexyl‐*N*‐(4‐((4‐(dimethylamino)‐6‐methoxypyrimidin‐2‐yl)amino)phenyl)propenamide (13)

General Procedure A was followed using **58** (17 mg, 0.07 mmol) and **68** (13 mg, 0.07 mmol) to afford **13** as a white powder (5 mg, 19%). ^1^H NMR (300 MHz, DMSO) *δ* 9.69 (s, 1H), 8.87 (s, 1H), 7.64 (d, *J* = 8.7 Hz, 2H), 7.42 (d, *J* = 8.9 Hz, 2H), 5.35 (s, 1H), 3.80 (s, 3H), 3.00 (s, 6H), 2.26 (t, *J* = 7.7 Hz, 2H), 1.75–1.60 (m, 5H), 1.47 (q, *J* = 7.3 Hz, 2H), 1.26–1.13 (m, 4H), 0.95–0.84 (m, 2H). ^13^C NMR (75 MHz, DMSO) *δ* 171.0, 170.4, 164.5, 158.6, 136.6, 132.8, 119.4, 118.9, 76.1, 53.0, 37.0, 36.9, 33.9, 32.8, 32.7, 26.2, 25.8. LCMS *m/z* 398.2 [M + H]^+^.

##### 3‐Cyclohexyl‐*N*‐(4‐((4‐(dimethylamino)‐6‐(trifluoromethyl)pyrimidin‐2‐yl)amino)phenyl)propenamide (14)

General Procedure A was followed using **58** (35 mg, 0.14 mmol) and **69** (32 mg, 0.14 mmol) to afford **14** as a white powder (30 mg, 48%). ^1^H NMR (300 MHz, DMSO) *δ* 9.74 (s, 1H), 9.41 (s, 1H), 7.61 (d, *J* = 9.0 Hz, 2H), 7.46 (d, *J* = 9.0 Hz, 2H), 6.44 (s, 1H), 3.11 (s, 6H), 2.27 (t, *J* = 7.7 Hz, 2H), 1.77–1.58 (m, 5H), 1.47 (q, *J* = 7.2 Hz, 2H), 1.26–1.12 (m, 4H), 0.96–0.79 (m, 2H). ^19^F NMR (282 MHz, DMSO) *δ* −69.3. LCMS *m/z* 436.2 [M + H]^+^.

##### 3‐Cyclohexyl‐*N*‐(4‐((4‐(dimethylamino)‐5,6,7,8‐tetrahydroquinazolin‐2‐yl)amino)phenyl)propenamide (15)

General Procedure A was followed using **58** (47 mg, 0.19 mmol) and **70** (40 mg, 0.19 mmol) to afford **15** as an off‐white powder (18 mg, 23%). ^1^H NMR (300 MHz, DMSO) *δ* 9.63 (s, 1H), 8.82 (s, 1H), 7.64 (d, *J* = 9.0 Hz, 2H), 7.40 (d, *J* = 8.9 Hz, 2H), 2.95 (s, 6H), 2.64–2.52 (m, 3H), 2.26 (t, *J* = 7.7 Hz, 2H), 1.79–1.58 (m, 9H), 1.48 (q, *J* = 7.3 Hz, 2H), 1.28–1.10 (m, 5H), 0.95–0.83 (m, 2H). ^13^C NMR (75 MHz, DMSO) *δ* 170.8, 165.8, 164.2, 156.6, 137.1, 132.2, 119.4, 118.1, 107.0, 40.4, 36.8, 33.9, 32.7, 32.6, 31.9, 26.1, 26.0, 25.8, 23.2, 22.1. LCMS *m/z* 422.2 [M + H]^+^.

##### 3‐Cyclohexyl‐*N*‐(4‐((4‐(dimethylamino)quinazolin‐2‐yl)amino)phenyl)propenamide (16)

General Procedure A was followed using **58** (26 mg, 0.11 mmol) and **75** (22 mg, 0.11 mmol) to afford **16** as an off‐white powder (11 mg, 25%). ^1^H NMR (300 MHz, DMSO) *δ* 9.72 (s, 1H), 9.11 (s, 1H), 8.00 (d, *J* = 8.3 Hz, 1H), 7.74 (d, *J* = 8.6 Hz, 2H), 7.60 (t, *J* = 7.3 Hz, 1H), 7.54–7.39 (m, 3H), 7.16 (t, *J* = 7.7 Hz, 1H), 3.31 (s, 6H), 2.28 (t, *J* = 7.7 Hz, 2H), 1.77–1.59 (m, 5H), 1.49 (q, *J* = 7.2 Hz, 2H), 1.26–1.12 (m, 4H), 0.89 (q, *J* = 11.3 Hz, 2H). ^13^C NMR (75 MHz, DMSO) *δ* 170.9, 163.6, 156.8, 155.2, 136.2, 133.2, 132.4, 126.3, 124.7, 120.8, 119.4, 119.3, 112.0, 41.7, 36.8, 33.9, 32.7, 32.6, 26.1, 25.8. LCMS *m/z* 418.0 [M + H]^+^.

##### 3‐Cyclohexyl‐*N*‐(4‐((6‐(dimethylamino)‐2‐methylpyrimidin‐4‐yl)amino)phenyl)propenamide (17)

General Procedure A was followed using **58** (35 mg, 0.14 mmol) and **71** (24 mg, 0.14 mmol) to afford **17** as an off‐white powder (32 mg, 59%). ^1^H NMR (300 MHz, DMSO) *δ* 9.72 (s, 1H), 8.78 (s, 1H), 7.59–7.29 (m, 4H), 5.61 (s, 1H), 2.96 (s, 6H), 2.36–2.18 (m, 5H), 1.77–1.55 (m, 5H), 1.48 (q, *J* = 7.3 Hz, 2H), 1.29–1.07 (m, 4H), 0.95–0.80 (m, 2H). ^13^C NMR (75 MHz, DMSO) *δ* 171.0, 165.3, 162.7, 161.0, 136.2, 133.4, 120.0, 119.7, 79.8, 36.8, 36.6, 33.9, 32.7, 32.6, 26.1, 26.0, 25.8. LCMS *m/z* 382.4 [M + H]^+^.

##### 3‐Cyclohexyl‐N‐(4‐((2‐(dimethylamino)‐6‐methylpyrimidin‐4‐yl)amino)phenyl)propenamide (18)

General Procedure A was followed using **58** (37 mg, 0.15 mmol) and **72** (26 mg, 0.15 mmol) to afford **18** as an orange powder (17 mg, 30%). ^1^H NMR (300 MHz, DMSO) *δ* 9.73 (s, 1H), 8.95 (s, 1H), 7.55 (d, *J* = 8.8 Hz, 2H), 7.47 (d, *J* = 8.7 Hz, 2H), 5.82 (s, 1H), 3.07 (s, 6H), 2.27 (t, *J* = 7.7 Hz, 2H), 2.11 (s, 3H), 1.76–1.62 (m, 5H), 1.48 (q, *J* = 7.2 Hz, 2H), 1.26–1.12 (m, 4H), 0.95–0.83 (m, 2H). ^13^C NMR (75 MHz, DMSO) *δ* 171.0, 164.5, 161.8, 160.7, 135.9, 133.4, 119.7, 119.5, 93.7, 36.8, 36.7, 33.9, 32.7, 32.6, 26.1, 25.8, 24.0. LCMS *m/z* 382.3 [M + H]^+^.

##### General Procedure B: 3‐Cyclohexyl‐*N*‐(4‐((6‐(dimethylamino)‐4‐methylpyridin‐2‐yl)amino)phenyl)propanamide (19)

**58** (40 mg, 0.16 mmol) Cs_2_CO_3_ (0.19 mmol), G3‐Pd‐xantphos (0.008 mmol), and **73** (28 mg, 0.16 mmol) were dissolved in 1,4‐dioxane (2 mL) and heated to 120 °C for 1 h using microwave irradiation. Upon completion, the reaction mixture was filtered through Celite, washed repeatedly with EtOAc, concentrated, and the crude residue was purified using flash column chromatography (0–5% MeOH/DCM) to afford **19** as an orange powder (22 mg, 35%). ^1^H NMR (300 MHz, DMSO) *δ* 9.62 (s, 1H), 8.39 (s, 1H), 7.51 (d, *J* = 8.6 Hz, 2H), 7.39 (d, *J* = 8.5 Hz, 2H), 6.04 (s, 1H), 5.80 (s, 1H), 2.89 (s, 6H), 2.31–2.16 (m, 5H), 1.76–1.58 (m, 5H), 1.48 (q, *J* = 7.5 Hz, 2H), 1.27–1.09 (m, 4H), 0.95–0.81 (m, 2H). ^13^C NMR (75 MHz, DMSO) *δ* 170.8, 156.4, 155.9, 155.4, 138.2, 131.8, 119.7, 118.1, 99.0, 88.0, 38.8, 36.8, 33.9, 32.7, 32.6, 26.1, 25.8, 24.5. LCMS *m/z* 381.2 [M + H]^+^.

##### 3‐Cyclohexyl‐*N*‐(4‐((4‐(dimethylamino)‐6‐methylpyridin‐2‐yl)amino)phenyl)propanamide (20)

General Procedure B was followed using **58** (65 mg, 0.26 mmol) and **74** (45 mg, 0.26 mmol) to afford **20** as an off‐white powder (26 mg, 26%). ^1^H NMR (300 MHz, DMSO) *δ* 9.63 (s, 1H), 8.41 (s, 1H), 7.50 (d, *J* = 8.6 Hz, 2H), 7.40 (d, *J* = 8.6 Hz, 2H), 6.05 (s, 1H), 5.80 (s, 1H), 2.89 (s, 6H), 2.31–2.19 (m, 5H), 1.75–1.57 (m, 5H), 1.48 (q, *J* = 7.3 Hz, 2H), 1.27–1.11 (m, 4H), 0.96–0.81 (m, 2H). ^13^C NMR (75 MHz, DMSO) *δ* 170.8, 156.3, 156.0, 155.1, 138.0, 131.9, 119.7, 118.2, 99.1, 87.9, 38.9, 36.8, 33.9, 32.7, 32.6, 26.1, 25.8, 24.4. LCMS *m/z* 381.2 [M + H]^+^.

##### 3‐Cyclohexyl‐*N*‐(4‐((1‐(dimethylamino)isoquinolin‐3‐yl)amino)phenyl)propanamide (21)

General Procedure B was followed using **58** (36 mg, 0.15 mmol) and **76** (30 mg, 0.15 mmol) to afford **21** as an orange powder (10 mg, 17%). ^1^H NMR (300 MHz, DMSO) *δ* 9.68 (s, 1H), 8.57 (s, 1H), 7.92 (d, *J* = 8.5 Hz, 1H), 7.55–7.38 (m, 6H), 7.19–7.11 (m, 1H), 6.53 (s, 1H), 3.06 (s, 6H), 2.29 (t, *J* = 7.7 Hz, 2H), 1.77–1.62 (m, 5H), 1.50 (q, *J* = 7.2 Hz, 2H), 1.21 (d, *J* = 17.6 Hz, 4H), 0.96–0.86 (m, 2H). ^13^C NMR (75 MHz, DMSO) *δ* 171.2, 160.7, 150.1, 141.0, 137.9, 132.4, 129.7, 126.1, 125.4, 121.3, 120.0, 118.7, 114.9, 93.2, 42.9, 37.0, 34.1, 32.9, 32.8, 26.3, 25.9. LCMS *m/z* 417.2 [M + H]^+^.

##### 3‐Cyclohexyl‐*N*‐(4‐((4‐(dimethylamino)quinolin‐2‐yl)amino)phenyl)propanamide (22)

General Procedure B was followed using **58** (20 mg, 0.10 mmol) and **77** (26 mg, 0.11 mmol) to afford **22** as a white powder (10 mg, 25%). ^1^H NMR (300 MHz, DMSO) *δ* 9.71 (s, 1H), 9.10 (s, 1H), 7.91–7.76 (m, 3H), 7.61 (dd, *J* = 8.4, 1.3 Hz, 1H), 7.55–7.42 (m, 3H), 7.21 (ddd, *J* = 8.2, 6.8, 1.4 Hz, 1H), 6.44 (s, 1H), 2.90 (s, 6H), 2.29 (t, *J* = 7.7 Hz, 2H), 1.74–1.62 (m, 4H), 1.50 (q, *J* = 7.2 Hz, 2H), 1.26–1.11 (m, 5H), 0.96–0.84 (m, 2H). ^13^C NMR (75 MHz, DMSO) *δ* 170.9, 157.4, 154.9, 148.7, 137.2, 132.7, 128.7, 127.0, 123.9, 121.2, 119.6, 119.2, 118.5, 99.2, 43.5, 36.8, 33.9, 32.7, 32.6, 26.1, 25.8. LCMS *m/z* 417.2 [M + H]^+^.

##### 3‐Cyclohexyl‐*N*‐(4‐((4‐(dimethylamino)‐6‐methylpyrimidin‐2‐yl)amino)‐3‐fluorophenyl)propenamide (23)

To a degassed stirred solution of **78** (56 mg, 0.19 mmol) in methanol (5 mL) was added a catalytic amount of palladium on carbon under N_2_. The reaction was evacuated and backfilled with H_2_ and stirred vigorously for 1 h. Upon completion, the mixture was filtered through Celite, washed with EtOAc repeatedly, and concentrated to give the reduced intermediate with sufficient purity as a dark solid (48 mg, 96%). General Procedure A was followed using this aniline intermediate (48 mg, 0.28 mmol) and **59** (74 mg, 0.28 mmol) to afford **23** as an off‐white powder (10 mg, 9%). ^1^H NMR (300 MHz, DMSO) *δ* 9.92 (s, 1H), 8.08 (s, 1H), 7.77 (t, *J* = 9.0 Hz, 1H), 7.57 (dd, *J* = 13.3, 2.3 Hz, 1H), 7.19 (dd, *J* = 8.4, 2.1 Hz, 1H), 5.97 (s, 1H), 2.97 (s, 6H), 2.29 (t, *J* = 7.7 Hz, 2H), 2.14 (s, 3H), 1.73–1.57 (m, 5H), 1.48 (q, *J* = 7.3 Hz, 2H), 1.25–1.13 (m, 4H), 0.95–0.82 (m, 2H). ^19^F NMR (282 MHz, DMSO) *δ* −122.8. ^13^C NMR (75 MHz, DMSO) *δ* 171.4, 165.0, 163.0, 159.4, 153.8 (d, *J* = 242.3 Hz), 134.9 (d, *J* = 10.1 Hz), 124.3 (d, *J* = 3.0 Hz), 123.3 (d, *J* = 11.3 Hz), 114.1 (d, *J* = 2.9 Hz), 106.2 (d, *J* = 24.7 Hz), 93.0, 36.8, 36.5, 33.9, 32.6, 32.5, 26.1, 25.7, 23.7. LCMS *m/z* 400.2 [M + H]^+^.

##### 3‐Cyclohexyl‐*N*‐(4‐((4‐(dimethylamino)‐6‐methylpyrimidin‐2‐yl)amino)‐2‐fluorophenyl)propenamide (24)

To a degassed stirred solution of **79** (38 mg, 0.13 mmol) in MeOH (5 mL) was added a catalytic amount of palladium on carbon under N_2_. The reaction was evacuated and backfilled with H_2_ and stirred vigorously for 1 h. Upon completion, the mixture was filtered through Celite, washed with EtOAc repeatedly, and concentrated to give the reduced intermediate with sufficient purity as a dark solid (26 mg, 76%). General Procedure A was followed using this intermediate (26 mg, 0.15 mmol) and **59** (40 mg, 0.15 mmol) to afford **24** as an off‐white powder (8 mg, 13%). ^1^H NMR (300 MHz, DMSO) *δ* 9.42 (s, 1H), 9.27 (s, 1H), 7.82 (d, *J* = 13.8 Hz, 1H), 7.52 (t, *J* = 9.0 Hz, 1H), 7.36 (d, *J* = 9.1 Hz, 1H), 6.09 (s, 1H), 3.07 (s, 6H), 2.32 (t, *J* = 7.9 Hz, 2H), 2.21 (s, 3H), 1.79–1.59 (m, 5H), 1.47 (q, *J* = 7.3 Hz, 2H), 1.28–1.13 (m, 5H), 0.95–0.82 (m, 2H). ^19^F NMR (282 MHz, DMSO) *δ* −122.9. ^13^C NMR (75 MHz, DMSO) *δ* 171.6, 162.7, 155.7, 152.5, 138.3 (d, *J* = 10.4 Hz), 125.2 (d, *J* = 3.0 Hz), 119.0 (d, *J* = 10.9 Hz), 114.2 (d, *J* = 2.9 Hz), 105.8 (d, *J* = 25.6 Hz), 93.7, 37.1, 36.7, 33.2, 32.7, 32.6, 26.1, 25.8, 22.6. Note: CF carbon signal too weak to observe in ^13^C NMR. LCMS *m/z* 400.2 [M + H]^+^.

##### 3‐Cyclohexyl‐N‐(6‐((4‐(dimethylamino)‐6‐methylpyrimidin‐2‐yl)amino)pyridin‐3‐yl)propenamide (25)

To a degassed stirred solution of **81** (150 mg, 0.55 mmol) in MeOH (8 mL) was added a catalytic amount of palladium on carbon under N_2_. The reaction was evacuated and backfilled with H_2_ and stirred vigorously for one hour. Upon completion, the mixture was filtered through Celite, washed with EtOAc repeatedly, and concentrated to give the reduced intermediate as a solid (94 mg, 70%). This intermediate (45 mg, 0.18 mmol), 3‐cyclohexylpropanoic acid (29 mg, 0.18 mmol), *N, N*‐diisopropylethylamine (64 µL, 0.37 mmol), and propyl phosphonic anhydride (165 µL, 0.37 mmol, 50% solution in EtOAc) were added to a microwave vial equipped with a stirring bar and dissolved in DCE (3 mL). The resultant mixture was heated at 130 °C for 1 h under microwave irradiation. The mixture was diluted with DCM (20 mL) and water (10 mL), the organic fraction was separated and further washed with brine (10 mL) before being dried over MgSO_4_, filtered and concentrated under reduced pressure. The crude residue was then purified using flash column chromatography (0‐5% MeOH/DCM) to afford **25** as a white powder (12 mg, 17%). ^1^H NMR (300 MHz, DMSO) *δ* 9.91 (s, 1H), 9.00 (bs, 1H), 8.42 (d, *J* = 2.5 Hz, 1H), 8.14 (t, *J* = 4.9 Hz, 1H), 7.91 (dd, *J* = 9.0, 2.5 Hz, 1H), 6.12 (s, 1H), 3.06 (s, 6H), 2.30 (t, *J* = 7.7 Hz, 2H), 2.23 (s, 3H), 1.74–1.60 (m, 5H), 1.49 (q, *J* = 6.9 Hz, 2H), 1.28–1.11 (m, 4H), 0.95–0.86 (m, 2H). ^13^C NMR (75 MHz, DMSO) *δ* 177.8, 171.5, 163.1, 162.7, 148.9, 138.5, 130.1, 129.0, 112.3, 94.2, 37.0, 36.8, 33.7, 32.6, 30.3, 26.1, 25.8, 23.2. LCMS *m/z* 383.2 [M + H]^+^.

##### 3‐Cyclohexyl‐*N*‐(5‐((4‐(dimethylamino)‐6‐methylpyrimidin‐2‐yl)amino)pyridin‐2‐yl)propenamide (26)

To a degassed stirred solution of **80** (100 mg, 0.36 mmol) in methanol (8 mL) was added a catalytic amount of palladium on carbon under N_2_. The reaction was evacuated and backfilled with H_2_ and stirred vigorously for 1 h. Upon completion, the mixture was filtered through Celite, washed with EtOAc repeatedly, and concentrated to give the reduced intermediate with sufficient purity as a colored solid (85 mg, 96%). General Procedure A was followed using this intermediate (85 mg, 0.34 mmol) and **59** (59 mg, 0.34 mmol) to afford **26** as an off‐white powder (24 mg, 18%). ^1^H NMR (300 MHz, DMSO) *δ* 10.19 (s, 1H), 9.08 (s, 1H), 8.63 (d, *J* = 2.7 Hz, 1H), 8.13 (dd, *J* = 9.0, 2.7 Hz, 1H), 7.95 (d, *J* = 9.0 Hz, 1H), 6.00 (s, 1H), 3.03 (s, 6H), 2.34 (t, *J* = 7.7 Hz, 2H), 2.18 (s, 3H), 1.74–1.55 (m, 5H), 1.47 (q, *J* = 7.3 Hz, 2H), 1.26–1.10 (m, 4H), 0.94–0.81 (m, 2H). ^13^C NMR (75 MHz, DMSO) *δ* 171.6, 165.0, 163.0, 159.0, 145.4, 137.9, 134.1, 127.4, 113.0, 93.1, 36.8, 36.7, 33.5, 32.6, 32.6, 26.1, 25.8, 23.7. LCMS *m/z* 383.2 [M + H]^+^.

##### 3‐Cyclohexyl‐*N*‐(4‐((4‐(piperazin‐1‐yl)quinazolin‐2‐yl)amino)phenyl)propanamide (55)

4N HCl in dioxane (1.07 mL, 4.30 mmol) was added slowly to a solution of **84** (24 mg, 0.04 mmol) in DCM (3 mL), and the mixture was left to react at room temperature overnight. On completion, the volatiles were removed under a stream of N_2,_ and the desired product was triturated from the crude residue using Et_2_O, filtered and washed with additional diethyl ether. This precipitate was then passed through an SCX column (with multiple MeOH washes followed by ammonia solution, 7 N in methanol to elute). The eluted fraction was then lyophilized to afford **55** as an off‐white solid (14 mg, 71%). ^1^H NMR (300 MHz, DMSO) *δ* 9.70 (s, 1H), 9.14 (s, 1H), 7.87–7.72 (m, 3H), 7.60 (t, *J* = 7.8 Hz, 1H), 7.53–7.42 (m, 3H), 7.17 (t, *J* = 7.6 Hz, 1H), 3.58 (t, *J* = 4.9 Hz, 4H), 2.91 (t, *J* = 4.4 Hz, 4H), 2.28 (t, *J* = 7.7 Hz, 2H), 1.78–1.58 (m, 5H), 1.49 (q, *J* = 7.3 Hz, 2H), 1.27–1.12 (m, 4H), 1.09 (t, *J* = 7.0 Hz, 1H), 0.95–0.84 (m, 2H). ^13^C NMR (75 MHz, DMSO) *δ* 170.9, 165.3, 155.9, 153.3, 136.5, 132.9, 132.5, 125.9, 125.4, 121.2, 119.4, 118.9, 112.4, 50.9, 45.5, 36.8, 33.9, 32.7, 32.6, 26.1, 25.8. LCMS *m/z* 459.2 [M + H]^+^.

##### 1‐(4‐Fluorophenyl)‐3‐(4‐((4‐(piperazin‐1‐yl)quinazolin‐2‐yl)amino)phenyl)urea (56)

4N HCl in dioxane (1.07 mL, 4.30 mmol) was added slowly to a solution of **85** (24 mg, 0.04 mmol) in DCM (3 mL), and the mixture was allowed to stir at room temperature for 20 h. On completion, the volatiles were removed under a stream of N_2,_ and the desired product was triturated from the crude residue using Et_2_O, filtered and washed with additional Et_2_O. This precipitate was then passed through an SCX column (with multiple MeOH washes followed by ammonia solution, 7 N in methanol to elute). The eluted fraction was then lyophilized to afford **56** as a yellow solid (12 mg, 61%). ^1^H NMR (300 MHz, DMSO) *δ* 8.88 (s, 1H), 8.64 (s, 1H), 8.44 (s, 1H), 7.65 (d, *J* = 8.8 Hz, 2H), 7.44 (dd, *J* = 9.1, 5.0 Hz, 2H), 7.30 (d, *J* = 8.9 Hz, 2H), 7.10 (t, *J* = 8.9 Hz, 2H), 5.98 (s, 1H), 3.05 (s, 6H), 2.18 (s, 3H). ^19^F NMR (282 MHz, DMSO) *δ* −121.9. ^13^C NMR (75 MHz, DMSO) *δ* 164.1, 162.9, 158.7, 157.2 (d, *J* = 237.7 Hz), 152.8, 136.3 (d, *J* = 2.1 Hz), 135.9, 132.8, 119.7 (d, *J* = 7.6 Hz), 119.1, 118.9, 115.2 (d, *J* = 22.0 Hz), 92.7, 36.9, 23.4. LCMS *m/z* 458.2 [M + H]^+^.

##### General Procedure C: *tert*‐Butyl (4‐(3‐cyclohexylpropanamido)phenyl)carbamate (57)

Oxalyl chloride (13 mmol) was added slowly to a stirred solution of 3‐cyclohexylpropanoic acid (2.03 g, 13.0 mmol) and DMF (1.3 mmol) in DCM (10 mL). After an hour of stirring at room temperature, the mixture was concentrated under N_2_ to give 3‐cyclohexylpropanoyl chloride as a crude residue. The residue was then redissolved in DCM (10 mL), and *tert*‐butyl (4‐aminophenyl)carbamate (3.69 g, 17.7 mmol) and triethylamine (177 mmol) were added, and the reaction mixture was allowed to stir at room temperature for 2 h. DCM (10 mL) was added to the mixture, and the solution was washed with saturated NaHCO_3_ and brine. The organic layer was then dried over MgSO_4_, filtered and concentrated under reduced pressure. The crude residue was then purified using flash column chromatography (0–100% EtOAc/heptane) to afford **57** as a white solid (3 g, 73%). ^1^H NMR (300 MHz, DMSO) *δ* 9.74 (s, 1H), 9.19 (s, 1H), 7.43 (d, *J* = 9.0 Hz, 2H), 7.32 (d, *J* = 8.9 Hz, 2H), 2.26 (t, *J* = 7.7 Hz, 2H), 1.72–1.47 (m, 6H), 1.45 (s, 9H), 1.32–1.00 (m, 5H), 0.95–0.79 (m, 2H). LCMS *m/z* 347.2 [M + H]^+^.

##### *N*‐(4‐Aminophenyl)‐3‐cyclohexylpropanamide (58)

Trifluoroacetic acid (662 µL, 8.66 mmol) was added slowly to a solution of **57** (300 mg, 0.87 mmol) in DCM (4 mL), and the mixture was allowed to react at room temperature. Complete conversion was observed after 2 h. The volatiles were removed under a stream of N_2_ to give the crude residue. This residue was dissolved in saturated NaHCO_3_ (15 mL), extracted with DCM (2 x 20 mL), and the combined organic fractions further washed with brine (10 mL) before being dried over MgSO_4_, filtered and concentrated under reduced pressure to afford **58** as an off‐white solid (138 mg, 65%). ^1^H NMR (300 MHz, DMSO) *δ* 9.42 (s, 1H), 7.18 (d, *J* = 8.7 Hz, 2H), 6.47 (d, *J* = 8.7 Hz, 2H), 4.81 (s, 2H), 2.21 (t, 2H), 1.75–1.55 (m, 5H), 1.45 (q, *J* = 7.1 Hz, 2H), 1.25–1.06 (m, 4H), 0.88 (m, 2H). LCMS *m/z* 247.2 [M + H]^+^.

##### General Procedure D: 2‐Chloro‐*N*,*N*,6‐trimethylpyrimidin‐4‐amine (59) and 4‐Chloro‐*N*,*N*,6‐trimethylpyrimidin‐2‐amine (72)

2,4‐Dichloro‐6‐methylpyrimidine (500 mg, 3.07 mmol) and dimethylamine (551 µL, 3.07 mmol, 33% solution in EtOH) were dissolved in DMF (3 mL). *N, N*‐diisopropylethylamine (6.14 mmol) was then added, and the reaction mixture was stirred at room temperature overnight. The mixture was concentrated to dryness in vacuo, and the crude residue was purified using flash column chromatography (0–100% EtOAc/heptane) to afford **59** as a white solid (254 mg, 48%) and **72** as a white solid (73 mg, 14%). **59**: ^1^H NMR (300 MHz, CDCl_3_) *δ* 6.2 (s, 1H), 3.1 (s, 6H), 2.3 (s, 3H). LCMS m/z 172.0 [M + H]^+^. **72**: ^1^H NMR (300 MHz, CDCl_3_) *δ* 6.4 (s, 1H), 3.2 (s, 6H), 2.3 (s, 3H). LCMS m/z 172.0 [M + H]^+^.

##### 2‐Chloro‐*N*‐ethyl‐*N*,6‐dimethylpyrimidin‐4‐amine (60)

General Procedure D was followed using 2,4‐dichloro‐6‐methylpyrimidine (250 mg, 1.53 mmol) and *N*‐methylethanamine (132 µL, 1.53 mmol) to afford **60** as a translucent oil (115 mg, 41%). ^1^H NMR (300 MHz, CDCl_3_) *δ* 6.1 (s, 1H), 3.6 (bs, 2H), 3.0 (s, 3H), 2.3 (s, 3H), 1.2 (t, *J* = 7.1 Hz, 3H). LCMS *m/z* 186.2 [M + H]^+^.

##### *N*‐Benzyl‐2‐chloro‐*N*,6‐dimethylpyrimidin‐4‐amine (61)

General Procedure D was followed using 2,4‐dichloro‐6‐methylpyrimidine (132 mg, 0.81 mmol) and *N*‐methyl‐1‐phenylmethanamine (105 µL, 0.81 mmol) to afford **61** as a white solid (140 mg, 70%). ^1^H NMR (300 MHz, CDCl_3_) *δ* 7.38–7.27 (m, 3H), 7.19 (d, *J* = 6.5 Hz, 2H), 6.19 (s, 1H), 4.77 (s, 2H), 3.05 (s, 3H), 2.33 (s, 3H). LCMS *m/z* 248.0 [M + H]^+^.

##### 2‐Chloro‐4‐methyl‐6‐(pyrrolidin‐1‐yl)pyrimidine (62)

General Procedure D was followed using 2,4‐dichloro‐6‐methylpyrimidine (200 mg, 1.23 mmol) and pyrrolidine (101 µL, 1.23 mmol) to afford **62** as a white solid (155 mg, 64%). ^1^H NMR (300 MHz, CDCl_3_) *δ* 6.0 (s, 1H), 3.6 (s, 2H), 2.3 (s, 3H), 2.0 (s, 4H), 3.3 (bs, 2H). LCMS *m/z* 198.2 [M + H]^+^.

##### 2‐Chloro‐4‐methyl‐6‐(piperidin‐1‐yl)pyrimidine (63)

General Procedure D was followed using 2,4‐dichloro‐6‐methylpyrimidine (150 mg, 0.92 mmol) and piperidine (91 µL, 0.92 mmol) to afford **63** as a white solid (102 mg, 53%). ^1^H NMR (300 MHz, CDCl_3_) *δ* 6.2 (s, 1H), 3.6–3.6 (m, 3H), 2.3 (s, 3H), 1.7–1.6 (m, 7H). LCMS *m/z* 212.2 [M + H]^+^.

##### 4‐(2‐Chloro‐6‐methylpyrimidin‐4‐yl)morpholine (64)

General Procedure D was followed using 2,4‐dichloro‐6‐methylpyrimidine (150 mg, 0.92 mmol) and morpholine (79 µL, 0.92 mmol) to afford **64** as a white solid (160 mg, 81%). ^1^H NMR (300 MHz, CDCl_3_) *δ* 6.2 (s, 1H), 3.8–3.7 (m, 4H), 3.7–3.6 (m, 4H), 2.3 (s, 3H). LCMS *m/z* 214.0 [M + H]^+^.

##### *tert*‐Butyl 4‐(2‐chloro‐6‐methylpyrimidin‐4‐yl)piperazine‐1‐carboxylate (65)

General Procedure D was followed using 2,4‐dichloro‐6‐methylpyrimidine (150 mg, 0.92 mmol) and *tert*‐butyl piperazine‐1‐carboxylate (171 mg, 0.92 mmol) to afford **65** as a white solid (118 mg, 41%). ^1^H NMR (300 MHz, CDCl_3_) *δ* 6.23 (s, 1H), 3.61 (d, *J* = 5.1 Hz, 4H), 3.50 (d, *J* = 3.3 Hz, 4H), 2.33 (s, 3H), 1.47 (s, 9H). LCMS *m/z* 313.2 [M + H]^+^.

##### 2‐Chloro‐4‐methyl‐6‐(4‐methylpiperazin‐1‐yl)pyrimidine (66)

General Procedure D was followed using 2,4‐dichloro‐6‐methylpyrimidine (150 mg, 0.92 mmol) and 1‐methylpiperazine (102 µL, 0.92 mmol) to afford **66** as a yellow solid (48 mg, 23%). ^1^H NMR (300 MHz, CDCl_3_) *δ* 6.2 (s, 1H), 3.7 (t, *J* = 5.2 Hz, 4H), 2.5 (t, *J* = 5.2 Hz, 4H), 2.3 (s, 6H). LCMS *m/z* 227.0 [M + H]^+^.

##### 2‐Chloro‐N,N‐dimethylpyrimidin‐4‐amine (67)

General Procedure D was followed using 2,4‐dichloropyrimidine (250 mg, 1.68 mmol) and dimethylamine (302 µL, 1.68 mmol, 33% solution in EtOH) to afford **67** as a translucent oil (103 mg, 39%). ^1^H NMR (300 MHz, CDCl_3_) *δ* 8.02 (d, *J* = 6.2 Hz, 1H), 6.32 (d, *J* = 6.2 Hz, 1H), 3.12 (s, 6H). LCMS *m/z* 158.2 [M + H]^+^.

##### 2‐Chloro‐6‐methoxy‐*N*,*N*‐dimethylpyrimidin‐4‐amine (68)

General Procedure D was followed using 2,4‐dichloro‐6‐methoxypyrimidine (400 mg, 2.23 mmol) and dimethylamine (402 µL, 2.23 mmol, 33% solution in EtOH) to afford **68** as a white solid (25 mg, 6%). ^1^H NMR (300 MHz, CDCl_3_) *δ* 5.58 (s, 1H), 3.90 (s, 3H), 3.05 (s, 6H). LCMS *m/z* 188.2 [M + H]^+^.

##### 2‐Chloro‐*N*,*N*‐dimethyl‐6‐(trifluoromethyl)pyrimidin‐4‐amine (69)

General Procedure D was followed using 2,4‐dichloro‐6‐(trifluoromethyl)pyrimidine (250 mg, 1.15 mmol) and dimethylamine (207 µL, 1.15  mmol, 33% solution in EtOH) to afford **69** as a white solid (84 mg, 32%). ^1^H NMR (300 MHz, CDCl_3_) *δ* 6.65 (s, 1H), 3.26 (s, 3H), 3.12 (s, 3H). LCMS *m/z* 226.2 [M + H]^+^.

##### 2‐Chloro‐*N*,*N*‐dimethyl‐5,6,7,8‐tetrahydroquinazolin‐4‐amine (70)

General Procedure D was followed using 2,4‐dichloro‐5,6,7,8‐tetrahydroquinazoline (100 mg, 0.49 mmol) and dimethylamine (106 µL, 0.59  mmol, 33% solution in EtOH) to afford **70** as an off‐white solid (76 mg, 73%). ^1^H NMR (300 MHz, CDCl_3_) *δ* 3.06 (s, 6H), 2.77 (t, *J* = 6.5 Hz, 2H), 2.58 (t, *J* = 5.9 Hz, 2H), 1.82 (p, *J* = 6.7 Hz, 2H), 1.68 (p, *J* = 6.1 Hz, 2H). LCMS *m/z* 212.2 [M + H]^+^.

##### 6‐Chloro‐*N*,*N*,2‐trimethylpyrimidin‐4‐amine (71)

General Procedure D was followed using 4,6‐dichloro‐2‐methylpyrimidine (300 mg, 1.84 mmol) and dimethylamine (165 µL, 0.92 mmol, 33% solution in EtOH) to afford **71** as a white solid (84 mg, 26%). ^1^H NMR (300 MHz, CDCl_3_) *δ* 6.25 (s, 1H), 3.10 (s, 6H), 2.50 (s, 3H). LCMS *m/z* 172.2 [M + H]^+^.

##### 6‐Chloro‐*N*,*N*,4‐trimethylpyridin‐2‐amine (73)

General Procedure D was followed using 2,6‐dichloro‐4‐methylpyridine (215 mg, 1.33 mmol) and dimethylamine (286 µL, 1.59 mmol, 33% solution in EtOH) to afford **73** as an orange solid (129 mg, 57%). ^1^H NMR (300 MHz, CDCl_3_) *δ* 6.28 (d, *J* = 2.2 Hz, 1H), 6.22 (d, *J* = 2.2 Hz, 1H), 2.93 (s, 6H), 2.35 (s, 3H). LCMS *m/z* 171.0 [M + H]^+^.

##### 2‐Chloro‐*N,N*,6‐trimethylpyridin‐4‐amine (74)

General Procedure D was followed using 2,4‐dichloro‐6‐methylpyridine (250 mg, 1.54 mmol) and dimethylamine (253 µL, 1.86 mmol, 33% solution in EtOH) to afford **74** as a white solid (162 mg, 62%). ^1^H NMR (300 MHz, CDCl_3_) *δ* 7.26 (d, *J* = 2.2 Hz, 1H), 7.18 (d, *J* = 2.2 Hz, 1H), 3.90 (s, 6H), 3.32 (s, 3H). LCMS *m/z* 171.0 [M + H]^+^.

##### 2‐Chloro‐*N*,*N*‐dimethylquinazolin‐4‐amine (75)

General Procedure D was followed using 2,4‐dichloroquinazoline (220 mg, 1.11 mmol) and dimethylamine (199 µL, 1.11 mmol, 33% solution in EtOH) to afford **75** as an off‐white solid (22 mg, 10%). ^1^H NMR (300 MHz, CDCl_3_) *δ* 8.01 (dd, *J* = 8.5, 1.6 Hz, 1H), 7.79 (dd, *J* = 8.4, 1.5 Hz, 1H), 7.69 (ddd, *J* = 8.4, 6.9, 1.3 Hz, 1H), 7.39 (ddd, *J* = 8.4, 6.9, 1.4 Hz, 1H), 3.42 (s, 6H). LCMS *m/z* 208.0 [M + H]^+^.

##### 3‐Chloro‐*N,N*‐dimethylisoquinolin‐1‐amine (76)

General Procedure D was followed using 1,3‐dichloroisoquinoline (125 mg, 0.63 mmol) and dimethylamine (136 µL, 0.76 mmol, 33% solution in EtOH) to afford **76** as a white solid (72 mg, 55%). ^1^H NMR (300 MHz, CDCl_3_) *δ* 8.05 (dd, *J* = 8.4, 1.1 Hz, 1H), 7.64–7.51 (m, 2H), 7.42 (ddd, *J* = 8.3, 6.5, 1.6 Hz, 1H), 7.13 (d, *J* = 0.9 Hz, 1H), 3.15 (s, 6H). LCMS *m/z* 207.2 [M + H]^+^.

##### 2‐Chloro‐*N*,*N*‐dimethylquinolin‐4‐amine (77)

General Procedure D was followed using 2,4‐dichloroquinoline (100 mg, 0.51 mmol) and dimethylamine (109 µL, 0.61 mmol, 33% solution in EtOH) to afford **77** as a white solid (44 mg, 42%). ^1^H NMR (300 MHz, CDCl_3_) *δ* 8.00 (d, *J* = 8.3 Hz, 1H), 7.96 (d, *J* = 8.5 Hz, 1H), 7.64 (ddd, *J* = 8.3, 7.0, 1.5 Hz, 1H), 7.45 (td, *J* = 7.4, 1.3 Hz, 1H), 6.71 (s, 1H), 3.07 (s, 6H). LCMS *m/z* 207.0 [M + H]^+^.

##### 3‐Cyclohexyl‐*N*‐(3‐fluoro‐4‐nitrophenyl)propenamide (78)

General Procedure C was followed using 3‐cyclohexylpropanoic acid (338 mg, 2.17 mmol) and 3‐fluoro‐4‐nitro‐aniline (461 mg, 2.95 mmol) to afford **78** as a yellow solid (106 mg, 18%). ^1^H NMR (300 MHz, CDCl_3_) *δ* 8.07 (t, *J* = 8.7 Hz, 1H), 7.83 (dd, *J* = 13.3, 2.3 Hz, 1H), 7.43 (s, 1H), 7.22 (ddd, *J* = 9.2, 2.3, 1.2 Hz, 1H), 2.46–2.38 (m, 2H), 1.76–1.68 (m, 5H), 1.67–1.60 (m, 3H), 1.32–1.14 (m, 5H), 0.99–0.87 (m, 3H). LCMS *m/z* 295.2 [M + H]^+^.

##### 3‐Cyclohexyl‐*N*‐(2‐fluoro‐4‐nitrophenyl)propenamide (79)

General Procedure C was followed using 3‐cyclohexylpropanoic acid (338 mg, 2.17 mmol) and 2‐fluoro‐4‐nitro‐aniline (461 mg, 2.95 mmol) to afford **79** as an off‐white solid (122 mg, 21%). ^1^H NMR (300 MHz, CDCl_3_) *δ* 8.66 (dd, *J* = 9.1, 7.8 Hz, 1H), 8.08 (dt, *J* = 9.3, 1.8 Hz, 1H), 8.00 (dd, *J* = 10.8, 2.5 Hz, 1H), 7.54 (s, 1H), 2.48 (t, *J* = 7.0 Hz, 2H), 1.78–1.70 (m, 3H), 1.65 (dd, *J* = 15.3, 7.2 Hz, 3H), 1.55 (s, 2H), 1.34–1.17 (m, 3H), 1.02–0.88 (m, 2H). LCMS *m/z* 295.1 [M + H]^+^.

##### 3‐Cyclohexyl‐*N*‐(5‐nitropyridin‐2‐yl)propenamide (80)

5‐Nitropyridin‐2‐amine (178 mg, 1.28 mmol), 3‐cyclohexylpropanoic acid (200 mg, 1.28 mmol), *N, N‐*diisopropylethylamine (446 µL, 2.56  mmol), and propyl phosphonic anhydride (1.15 mL, 2.56 mmol, 50% solution in EtOAc) were added to a microwave vial equipped with a stirring bar and dissolved in DCE (3 mL). The resultant mixture was heated at 130 °C for 1 h using microwave irradiation. The mixture was diluted with DCM (20 mL) and water (10 mL), the organic fraction was separated and further washed with brine (10 mL) before being dried over MgSO_4_, filtered and concentrated under reduced pressure. The crude residue was then purified using flash column chromatography (0‐100% EtOAc/heptane) to afford **80** as a white solid (292 mg, 82%). ^1^H NMR (300 MHz, CDCl_3_) *δ* 9.1 (s, 1H), 8.7 (s, 1H), 8.5 (d, *J* = 9.2 Hz, 1H), 8.4 (d, *J* = 9.3 Hz, 1H), 2.5 (t, *J* = 7.8 Hz, 2H), 1.8–1.6 (m, 7H), 1.3–1.1 (m, 4H), 1.0–0.8 (m, 2H). LCMS *m/z* 278.0 [M + H]^+^.

##### *N* ^4^,*N* ^4^,6‐Trimethyl‐*N* ^2^‐(5‐nitropyridin‐2‐yl)pyrimidine‐2,4‐diamine (81)

General Procedure A was followed using **59** (300 mg, 1.75 mmol) and 5‐nitropyridin‐2‐amine (365 mg, 2.62 mmol) to afford **81** as a yellow residue (91 mg, 19%). ^1^H NMR (300 MHz, CDCl_3_) *δ* 9.19 (s, 1H), 8.54–8.35 (m, 2H), 6.04 (s, 1H), 3.26 (s, 6H), 2.46 (s, 3H). Note: Exchangeable proton NH not observed in ^1^H NMR. LCMS *m/z* 275.2 [M + H]^+^.

##### *tert*‐Butyl 4‐(2‐chloroquinazolin‐4‐yl)piperazine‐1‐carboxylate (82)

General Procedure D was followed using 2,4‐dichloroquinazoline (400 mg, 2.01 mmol) and *tert*‐butyl piperazine‐1‐carboxylate (445 mg, 2.42  mmol) to afford **82** as a white solid (425 mg, 61%). ^1^H NMR (300 MHz, CDCl_3_) *δ* 7.86 (dd, *J* = 3.7, 1.4 Hz, 1H), 7.83 (dd, *J* = 3.7, 1.4 Hz, 1H), 7.74 (ddd, *J* = 8.4, 6.8, 1.3 Hz, 1H), 7.45 (ddd, *J* = 8.3, 6.8, 1.4 Hz, 1H), 3.84 (dd, *J* = 6.8, 3.7 Hz, 4H), 3.74–3.55 (m, 4H), 1.49 (s, 9H). LCMS *m/z* 349.0 [M + H]^+^.

##### *tert*‐Butyl 4‐(2‐((4‐aminophenyl)amino)quinazolin‐4‐yl)piperazine‐1‐carboxylate (83)

General Procedure B was followed using **82** (100 mg, 0.29 mmol) and 4‐nitroaniline (59 mg, 0.43 mmol) to afford the intermediate as a yellow oil (121 mg, 94%). This intermediate (120 mg, 0.27 mmol) was dissolved in MeOH (10 mL), and a catalytic amount of palladium on carbon was added under N_2_. The reaction was evacuated and backfilled with H_2_ and stirred vigorously for 1 h. The crude residue was filtered through Celite, washed repeatedly with EtOAc, concentrated and purified using flash column chromatography (0–5% MeOH/DCM) to afford **83** as a yellow oil (94 mg, 84%). ^1^H NMR (300 MHz, CDCl_3_) *δ* 7.69 (d, *J* = 8.2 Hz, 1H), 7.55 (dd, *J* = 5.4, 1.6 Hz, 2H), 7.43 (d, *J* = 8.6 Hz, 2H), 7.12 (ddd, *J* = 8.2, 5.8, 2.5 Hz, 2H), 6.67 (d, *J* = 8.6 Hz, 2H), 3.62 (s, 8H), 1.49 (s, 9H). LCMS *m/z* 421.2 [M + H]^+^.

##### *tert*‐Butyl 4‐(2‐((4‐(3‐cyclohexylpropanamido)phenyl)amino)quinazolin‐4‐yl)piperazine‐1‐carboxylate (84)

General Procedure C was followed using 3‐cyclohexylpropanoic acid (30 mg, 0.19 mmol) and **83** (74 mg, 0.18 mmol) to afford **84** as a white solid (46 mg, 47%). ^1^H NMR (300 MHz, CDCl_3_) *δ* 7.91 (s, 1H), 7.76 (d, *J* = 8.3 Hz, 1H), 7.64 (dt, *J* = 15.6, 7.9 Hz, 2H), 7.56 (d, *J* = 8.8 Hz, 2H), 7.45 (d, *J* = 8.6 Hz, 2H), 3.89 (d, *J* = 5.8 Hz, 4H), 3.70–3.58 (m, 4H), 2.47–2.37 (m, 2H), 1.76–1.58 (m, 7H), 1.50 (s, 9H), 1.23 (dd, *J* = 22.1, 9.5 Hz, 4H), 0.92 (q, *J* = 10.8 Hz, 2H). LCMS *m/z* 559.2 [M + H]^+^.

##### *tert*‐Butyl 4‐(2‐((4‐(3‐(4‐fluorophenyl)ureido)phenyl)amino)quinazolin‐4‐yl)piperazine‐1‐carboxylate (85)

To a solution of **83** (50 mg, 0.12 mmol) in anhydrous THF (4 mL), 4‐nitrophenyl chloroformate (29 mg, 0.14 mmol) was added, and the reaction mixture was stirred at room temperature for 30 min. 4‐Fluoroaniline (0.02 mL, 0.20 mmol) and triethylamine (0.04 mL, 0.30 mmol) were then added and allowed to stir at room temperature for 20 h. Upon completion, THF was removed, and the crude material was dissolved in EtOAc and then washed repeatedly with saturated NaHCO_3_. The combined organic material was dried over MgSO_4_, filtered, concentrated under reduced pressure, and purified using flash column chromatography to afford **85** as a yellow solid (30 mg, 66%). ^1^H NMR (300 MHz, DMSO) *δ* 9.18 (s, 1H), 8.63 (s, 1H), 8.47 (s, 1H), 7.90–7.75 (m, 3H), 7.62 (t, *J* = 7.5 Hz, 1H), 7.54–7.43 (m, 3H), 7.35 (d, *J* = 8.9 Hz, 2H), 7.19 (t, *J* = 7.3 Hz, 1H), 7.11 (t, *J* = 8.9 Hz, 2H), 3.67–3.60 (m, 4H), 3.60–3.53 (m, 4H), 1.44 (s, 9H). LCMS *m/z* 558.2 [M + H]^+^.

##### Biology Experimental: *P. falciparum* 3D7 Asexual Stage Parasite Assay

This was performed as previously described, whereby lactate dehydrogenase (LDH) was used as a metabolic marker for parasite growth after a 72‐h incubation period.^[^ ^14^ ^,^ ^30^ ^]^

##### Human HepG2 Cell Viability Assay

This was performed as previously described.^[^ ^14^ ^,^ ^31^ ^]^

##### In Vitro ADME Assays

This was performed as previously described.^[^ ^14^ ^,^ ^16^ ^]^

##### Resistance Selection

This was performed as previously described.^[^ ^14^ ^]^ Briefly, 3D7 wildtype parasites initially exposed to 2 × EC_50_ (0.3 µM) of W482 (1) and incremental increases in EC_50_ were applied until 10 × EC_50_ was reached. The resistant populations were not cloned for whole‐genome sequencing.

##### Biology Experimental: Whole Genome Sequencing of W482‐Resistant Populations

This was performed as previously described.^[^ ^14^ ^]^ The data for this study have been deposited in the European Nucleotide Archive (ENA) at EMBL‐EBI under accession number PRJEB96799, with wildtype sequences at PRJEB58099.

##### Biology Experimental: Parasite Reduction Ratio Assay

This assay was conducted following a previously described protocol using the *Plasmodium falciparum* strain 3D7A, MRA‐151.^[^ ^19^ ^,^ ^28^ ^]^ The human biological samples used in the study were sourced ethically, and their research use was conducted in accordance with the terms of informed consent under an IRB/EC‐approved protocol.

##### Stage of Asexual Arrest Assay

This assay was performed as previously described with W482 (1) and DMSO being applied at 10 × EC_50_ (1.4 µM) and 0.04%, respectively.^[^ ^14^ ^]^

##### Parasite Proliferation Assays

Parasite proliferation assays were performed with PfABCI3‐HAreg parasites^[^ ^19^ ^]^ according to a previously described method, with the fluorescent nucleic acid intercalating dye SYBR Safe used to measure parasite viability.^[^ ^32^ ^,^ ^33^ ^]^ The assays were commenced with cultures consisting of predominantly ring‐stage parasites with a starting hematocrit of 1% and parasitemia of 1%. The duration of the assays was 72 h, and the concentration of DMSO in the assays did not exceed 0.1% v/v. It was shown previously that there is a reduction in *pfabci3‐ha* mRNA in PfABCI3‐HAreg exposed to 5 mM GlcN and a decrease in parasite viability over time.^[^ ^19^ ^]^ The level of knockdown of the protein is not known.

## Abbreviations

**Table:** 

CNV	copy number variation
GlcN	glucosamine
HA	hemagglutinin
HLM	human liver microsome
LCMS	liquid chromatography mass spectrometers
LDH	lactate dehydrogenase
*Pf*	*Plasmodium falciparum*
PRR	parasite reduction ratio
RHep	rat hepatocyte
SAR	structure‐activity relationship
SNP	single‐nucleotide polymorphisms
TCP	target candidate profile

## Supporting Information

Supporting Information file contains activity data on compounds against *P. falciparum* 3D7 and compound‐resistant parasites, asexual microscopy repeats, whole genome sequencing data, and compound spectra.

## Conflict of Interest

The authors declare no conflict of interest.

## Supporting information

Supplementary Material

## Data Availability

The data that support the findings of this study are available from the corresponding author upon reasonable request.
