# Supplementary material for: Exploration and Characterization of the Antimalarial Activity of Pyrimidine‐2,4‐Diamines for which Resistance is Mediated by the ABCI3 Transporter
Source: ChemMedChem. 2025 Dec 12;21(1):e202500739. doi: 10.1002/cmdc.202500739 (PMC12811996; doi:10.1002/cmdc.202500739)

SUPPORTING INFORMATION FOR

Exploration and Characterisation of the Antimalarial Activity of Pyrimidine-2,4-Diamines for Which Resistance is Mediated by the ABCI3 Transporter

Mahta Mansouri,[a,b] Madeline G. Dans,[a,b] Zijun Low,[a,b] Katie Loi,[a,b] Kate E. Jarman,[a,b] Jocelyn Penington,[a] Deyun Qiu,[c] Adele M. Lehane,[c] Benigno Crespo,[d] Francisco-Javier Gamo,[d] Delphine Baud,[e] Stephen Brand,[e] Paul F. Jackson,[f] Alan F. Cowman,[a,b] and Brad E. Sleebs.[a,b,*]

[a] The Walter and Eliza Hall Institute of Medical Research, Parkville 3052, Australia.

[b] Department of Medical Biology, The University of Melbourne, Parkville 3010, Australia.

[c] Research School of Biology, Australian National University, Canberra, 2601, Australia

[d] Global Health Medicines R &D, GSK, Tres Cantos, 28760, Spain

[e] MMV Medicines for Malaria Venture, 1215 Geneva, Switzerland.

[f] Emerging Science & Innovation, Discovery Sciences, Janssen R&D LLC, La Jolla, California 92121, USA.

[*] Correspondence to:
Brad E. Sleebs
The Walter and Eliza Hall Institute of Medical Research
1G Royal Parade, Parkville 3052, Victoria, Australia
Phone: 61 3 9345 2718
Email: sleebs@wehi.edu.au

Index

Page

S3	Figure S1	Dose response against *P. falciparum* 3D7 asexual parasites.
S4	Figure S2	Copy number analysis on W482-resistant parasite populations.
S5	Figure S3	Copy number on chromosome 3 of W482-resistant parasite populations.
S6	Table S1	Structural variant analysis on W482-resistant parasite populations.
S6	Table S2	CNV genes from W482-resistant parasite populations.
S7	Table S3	SNP analysis on W482-resistant parasite populations.
S8	Figure S4	Replicates of Giemsa-stained blood smears.
S9		LC chromatograms and NMR spectra of selected final compounds.

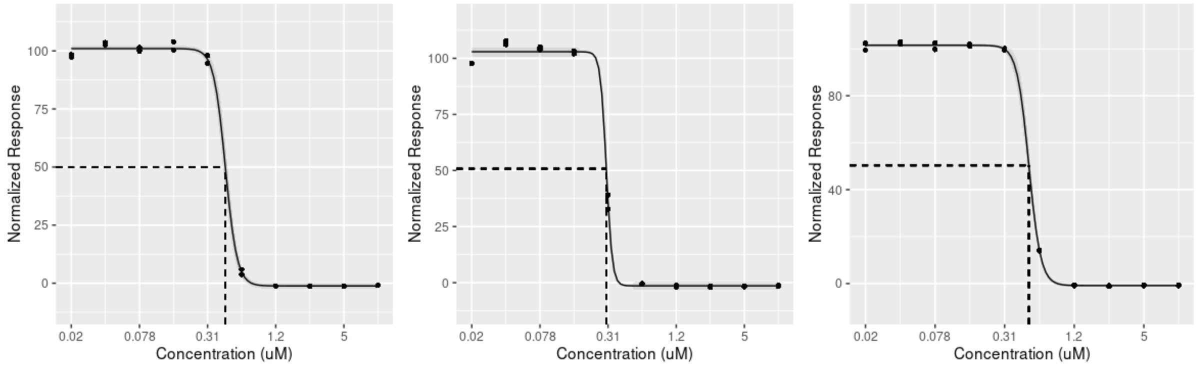

Figure S1. Dose response curves of W482 (**1**) against *P. falciparum* 3D7. Each dose response is from one experiment (performed in technical duplicates), measuring the LDH activity of *P. falciparum* 3D7 parasites following exposure to compounds for 72 h.

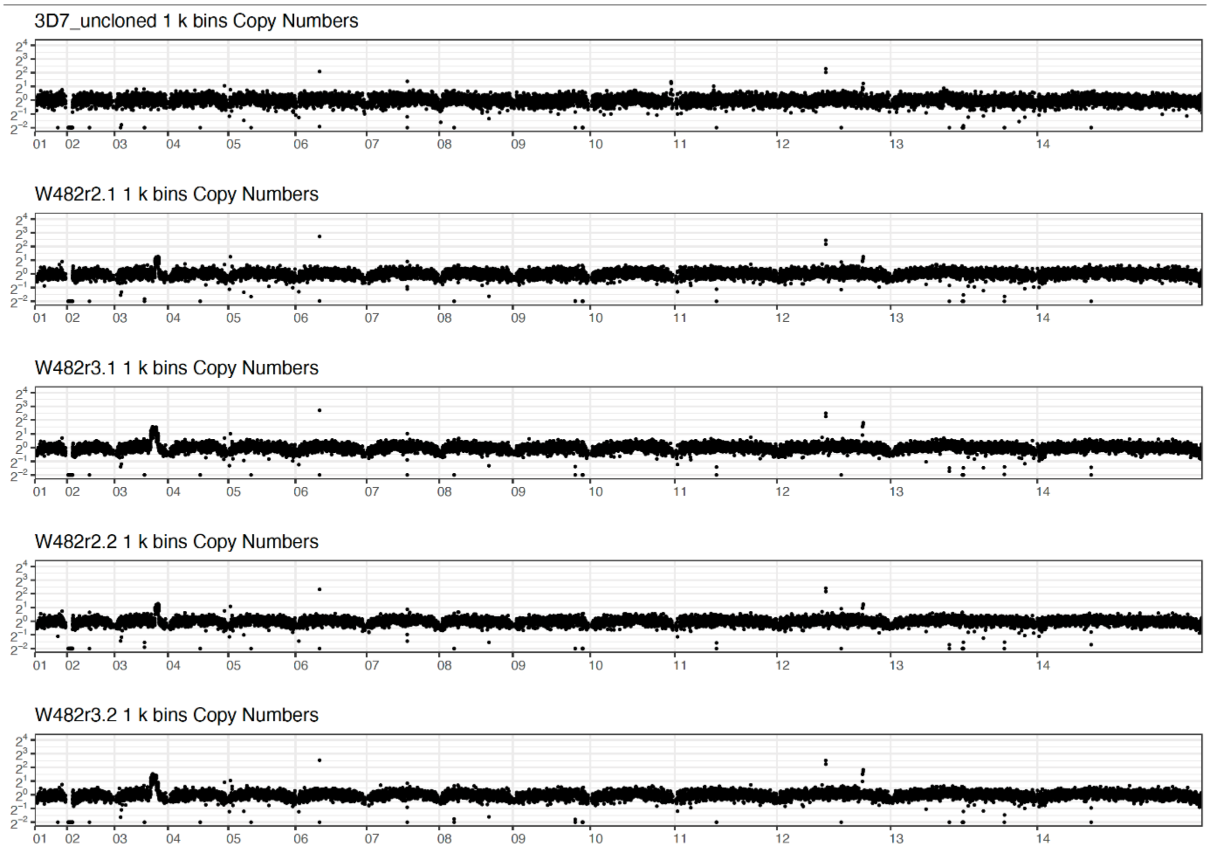

Figure S2. Copy number analysis shows duplication events on chromosome 3 in W482-resistant not present in 3D7 wildtype parasites. W482-resistant parasites and 3D7 wildtype parasites were aligned to the reference PlasmoDB-54_Pfalciparum3D7 using bwa-mem. Sequences were filtered using Picard Mark Duplicates, and quality was confirmed using the fastQC program. Copy number analysis was done with the QDNASeq R package using 1 kbp bins.

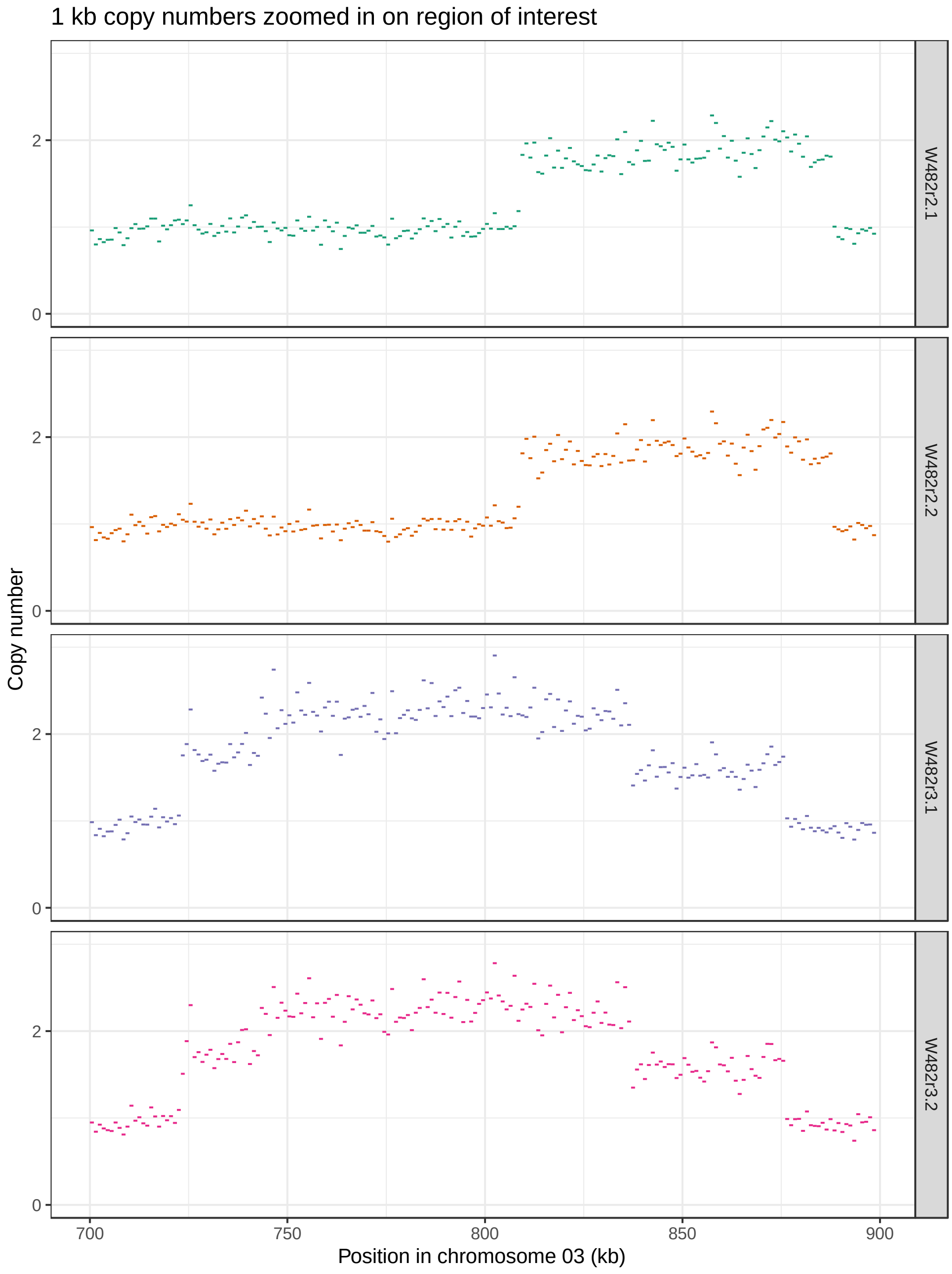

Figure S3. Copy number of W482-resistant populations copy numbers shown scaled relative to the wildtype sample. There were duplication events in chromosome 3, which were different but overlapping in the two pairs of samples.

Table S2. Structural variant events in chromosome 3 supporting the amplification from W482-resistant parasite populations (in duplicate).

GRIDSS ID	Position 1	Position 2	3D7	#1.1	#1.2	#2.1	#2.2
gridss0bf_33098	808,662	887,962	160	5069	5275	38	16
gridss0bf_31710	723,273	836,978	0	0	0	884	733
gridss0bf_32099	743,261	876,137	0	0	21	2424	2581

Table S3. Summary of the genes on chromosome 3 that exhibit a CNV (internal 808662 (population #1 copy number increases to 2) – 836978 (population #2 copy number drops below 2)) from whole genome sequencing of W482-resistant populations.

Gene ID	Gene Name	Description
PF3D7_0319500	–	RNA-binding protein, putative
PF3D7_0319600	EF-1delta	elongation factor 1-delta, putative
PF3D7_0319800	Unnamed	conserved *Plasmodium* protein, unknown function
PF3D7_0319700	ABCI3	ABC transporter I family member 1, putative
PF3D7_0319400	–	kinesin-8X
PF3D7_0319900	Unnamed	conserved protein, unknown function

Table S3. Summary of non-synonymous single-nucleotide polymorphisms identified from whole genome sequencing of W482-resistant populations #1 and #2. A T239A mutation in conserved protein LSA1 (PF3D7_1036400), as well as an I7040M mutation in an unnamed conserved *Plasmodium* protein with an unknown function.

**Chromosome**	**Position**	**Base change**	**Amino acid change**	**Gene ID**	**Gene description**
10	1437030	A→G	T239A	PF3D7_1036400	LSA1, conserved protein
13	191541	T→C	I7040M	PF3D7_1303800	conserved *Plasmodium* protein, unknown function

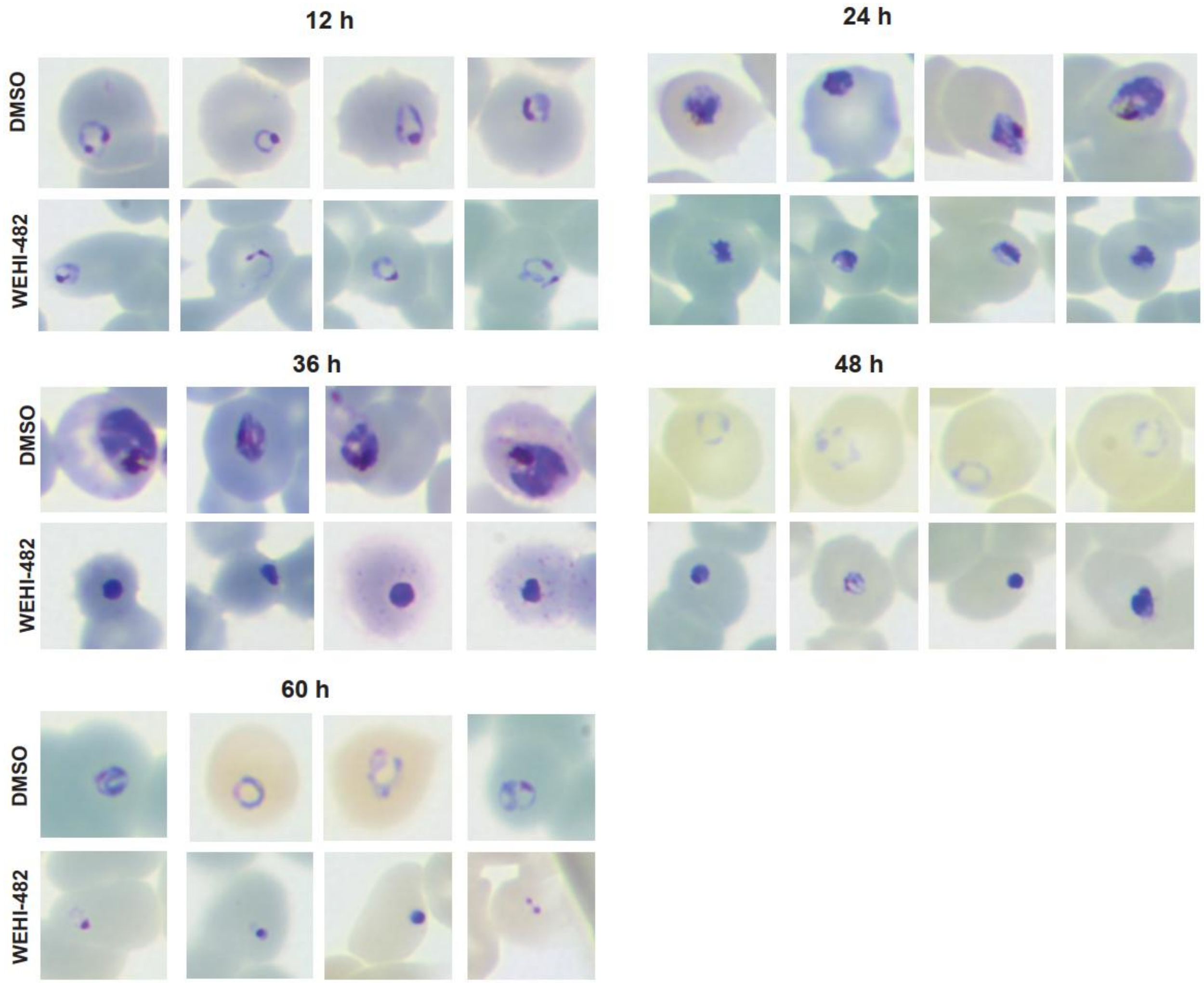

Figure S4. Giemsa-stained blood smears from each of the 3 technical replicates from the stage of arrest assay.

LC chromatograms and NMR spectra of selected final compounds

Compound **1**

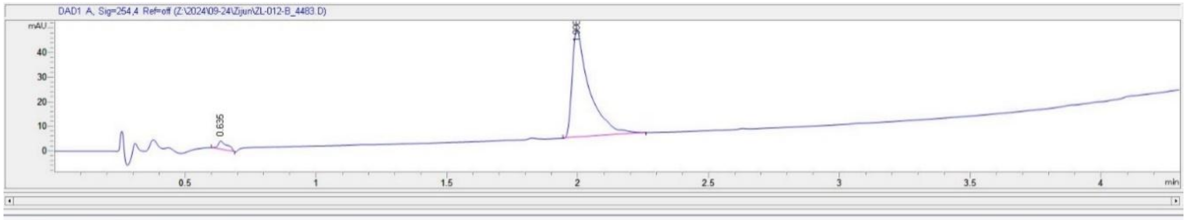

#	Time	Area	Height	Width	Area%	Symmetry
1	0.635	8.8	3.4	0.0347	4.148	0.367
2	1.996	203.4	44.3	0.0635	95.852	0.339

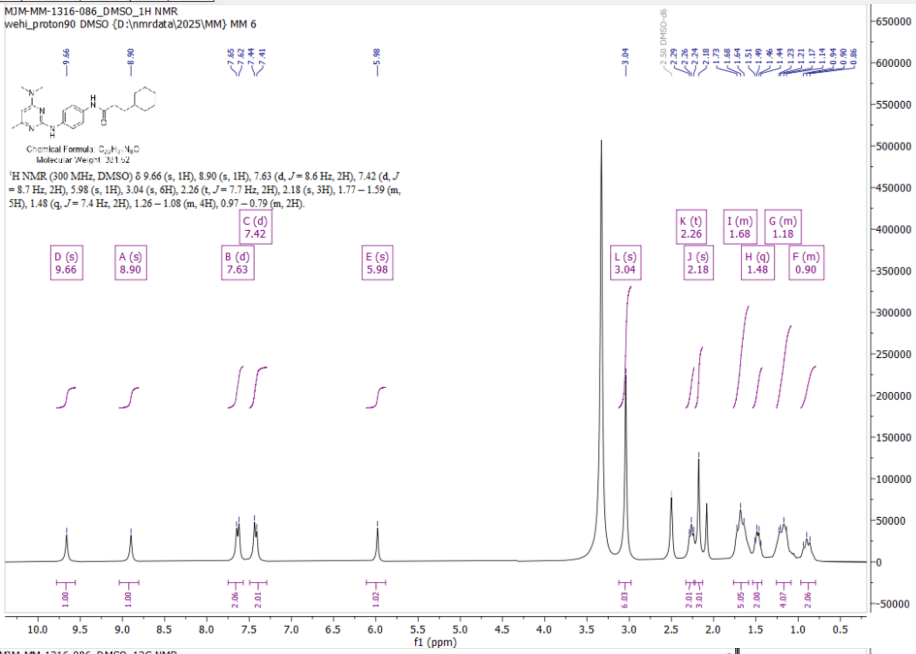

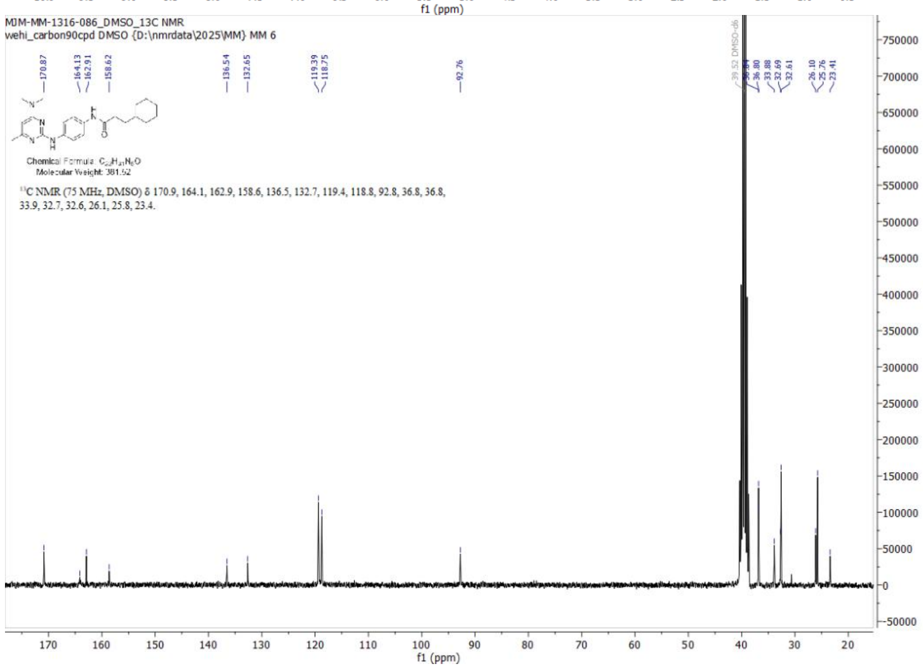

Compound **5**

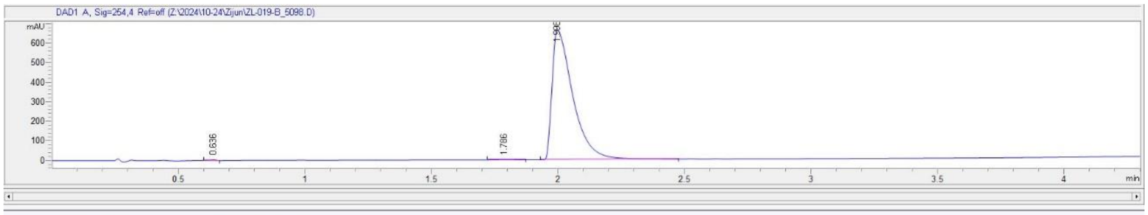

#	Time	Area	Height	Width	Area%	Symmetry
1	0.636	5.8	3.6	0.0266	0.152	0.784
2	1.786	4.2	1	0.0573	0.111	0.687
3	1.998	3790.2	668.3	0.0847	99.737	0.327

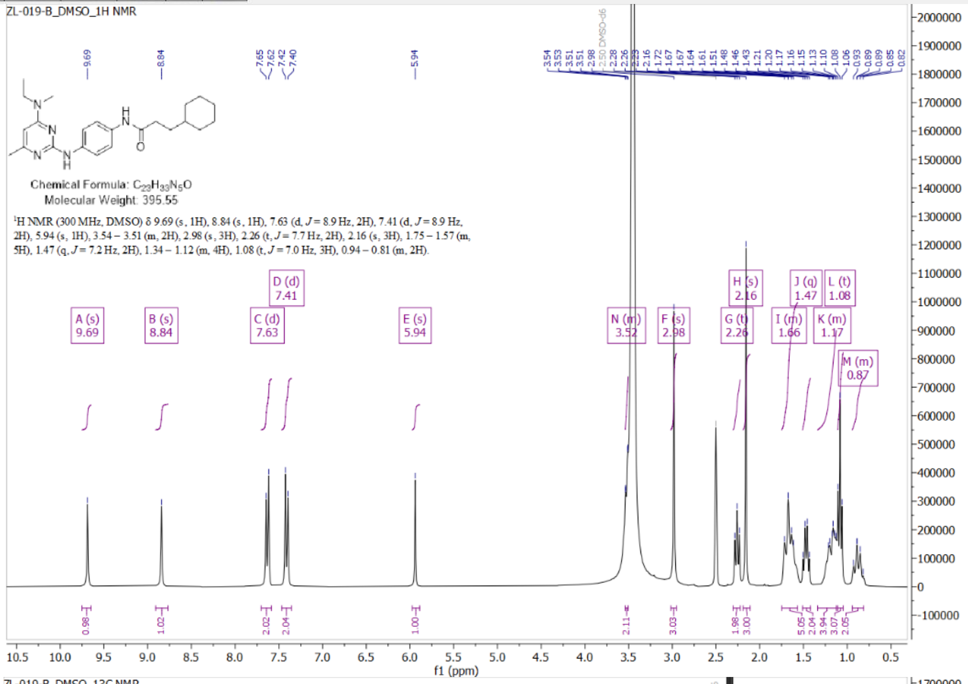

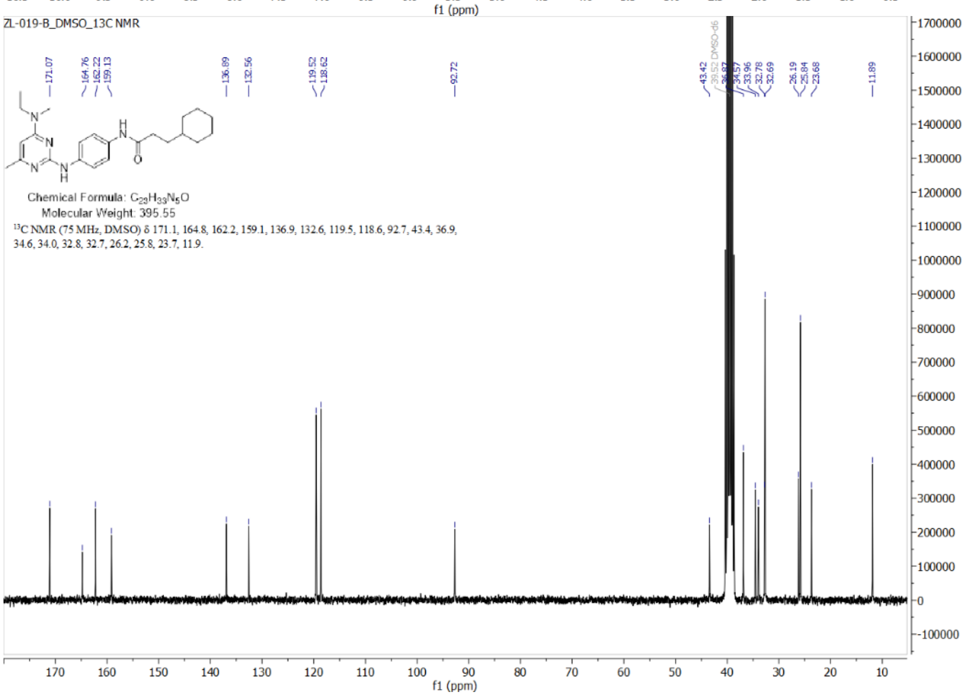

Compound **7**

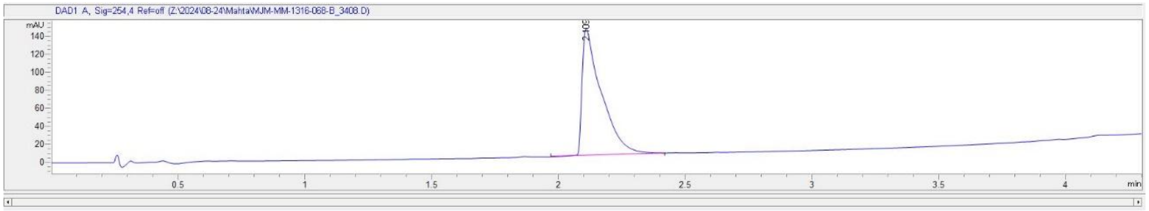

#	Time	Area	Height	Width	Area%	Symmetry
1	2.109	750.9	141.7	0.0728	100.000	0.249

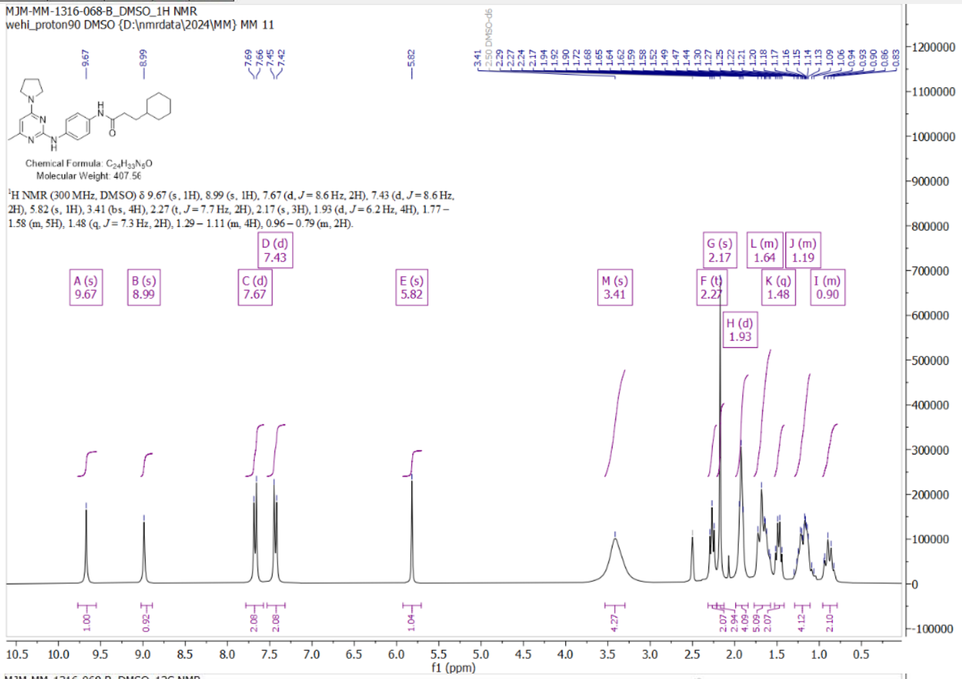

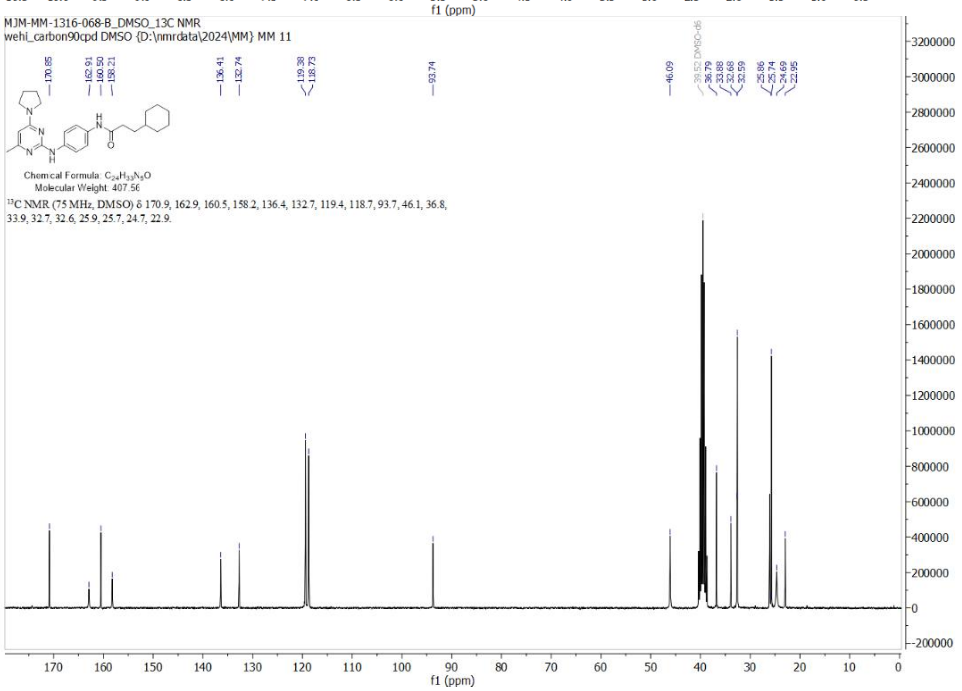

Compound **8**

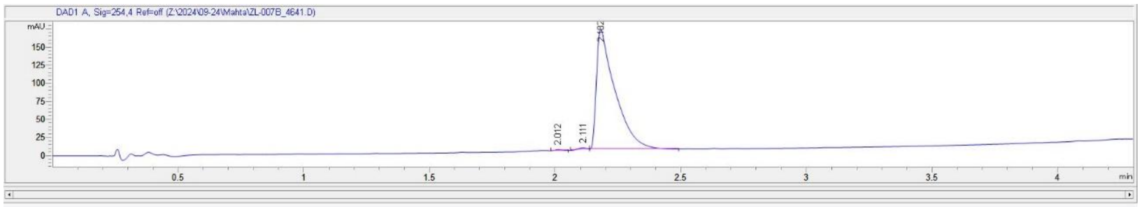

#	Time	Area	Height	Width	Area%	Symmetry
1	2.012	2.4	1.4	0.0261	0.272	0.794
2	2.111	5.9	2.4	0.041	0.679	1.645
3	2.182	857.9	166.4	0.0702	99.048	0.274

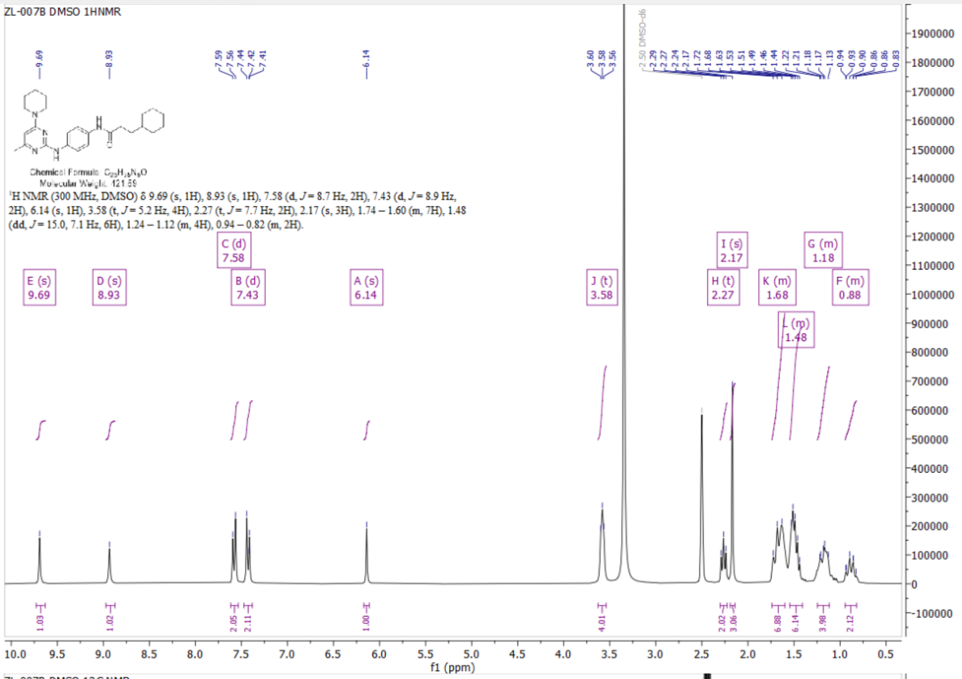

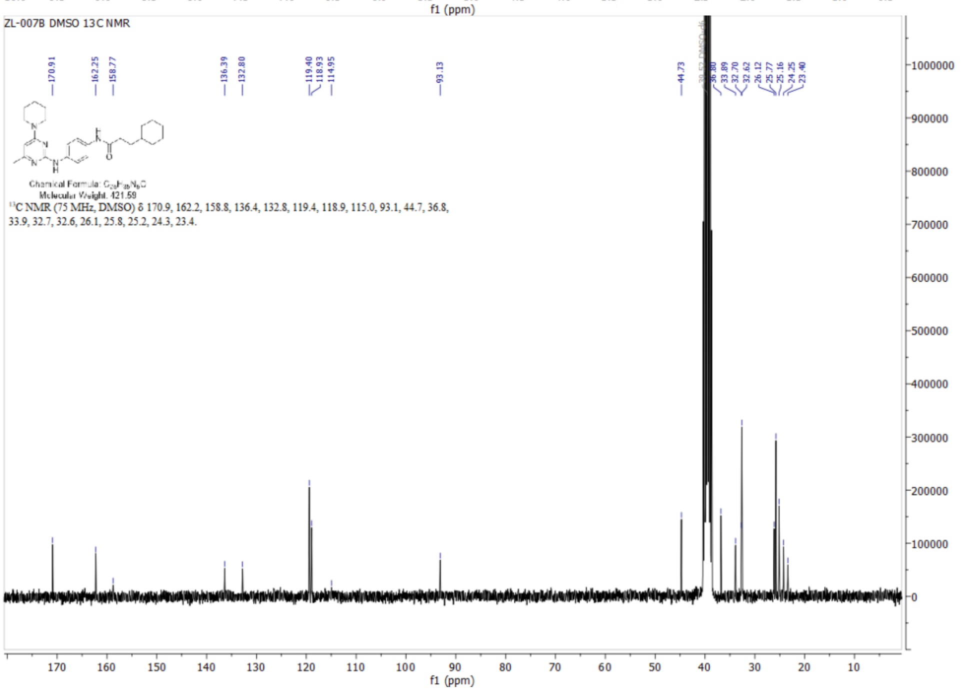

Compound **10**

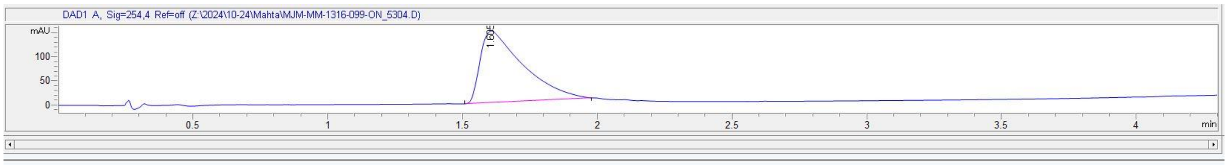

#	Time	Area	Height	Width	Area%	Symmetry
1	1.605	1650.2	150.3	0.1541	100.000	0.321

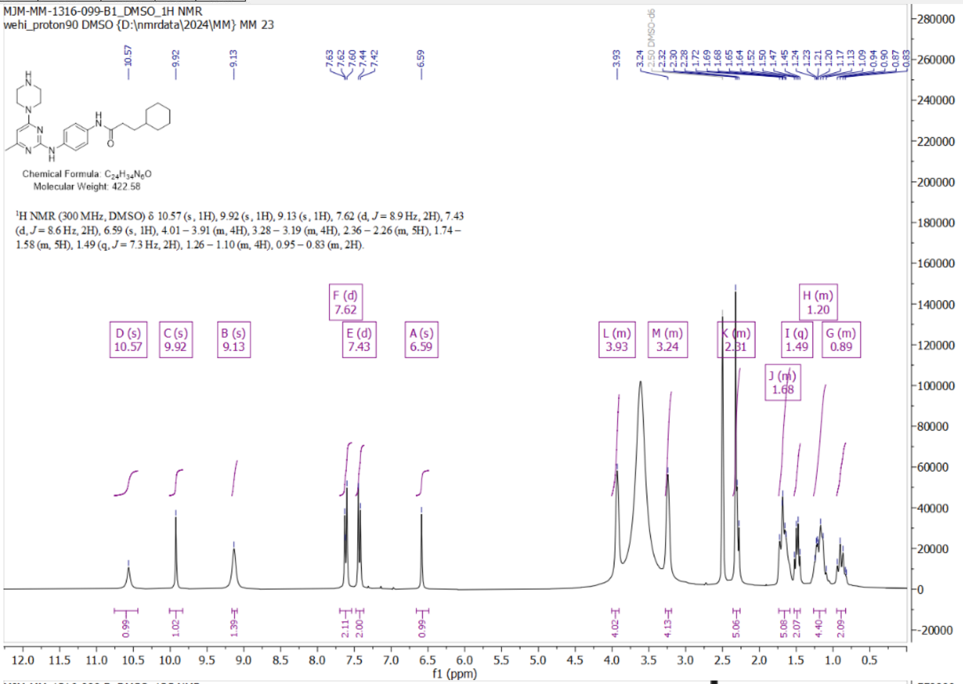

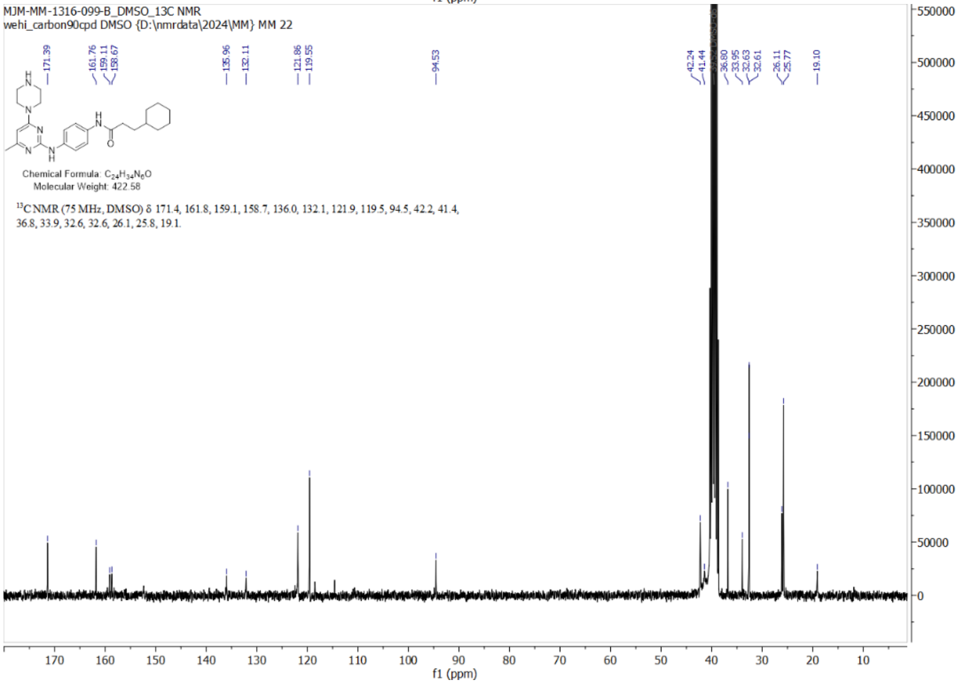

Compound **11**

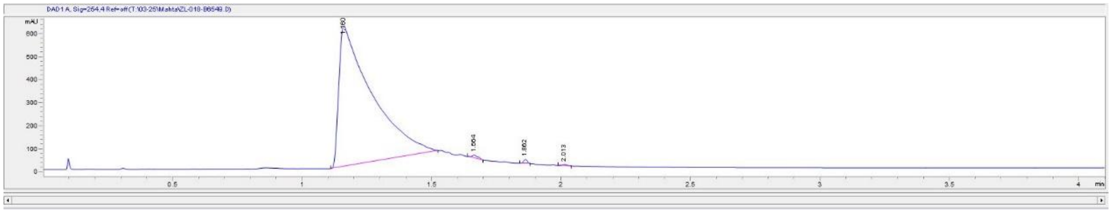

#	Time	Area	Height	Width	Area%	Symmetry
1	1.16	5404.5	611.8	0.1121	99.149	0.164
2	1.664	22.2	12.8	0.0237	0.406	0.503
3	1.862	17.5	17.8	0.0155	0.322	1.002
4	2.013	6.7	5.3	0.0204	0.123	0.847

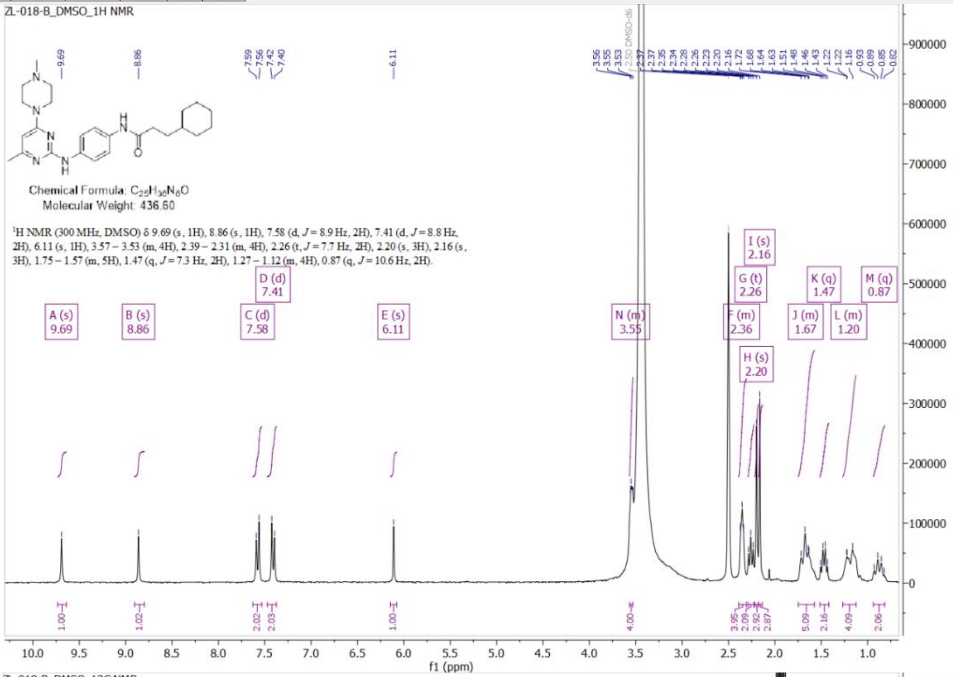

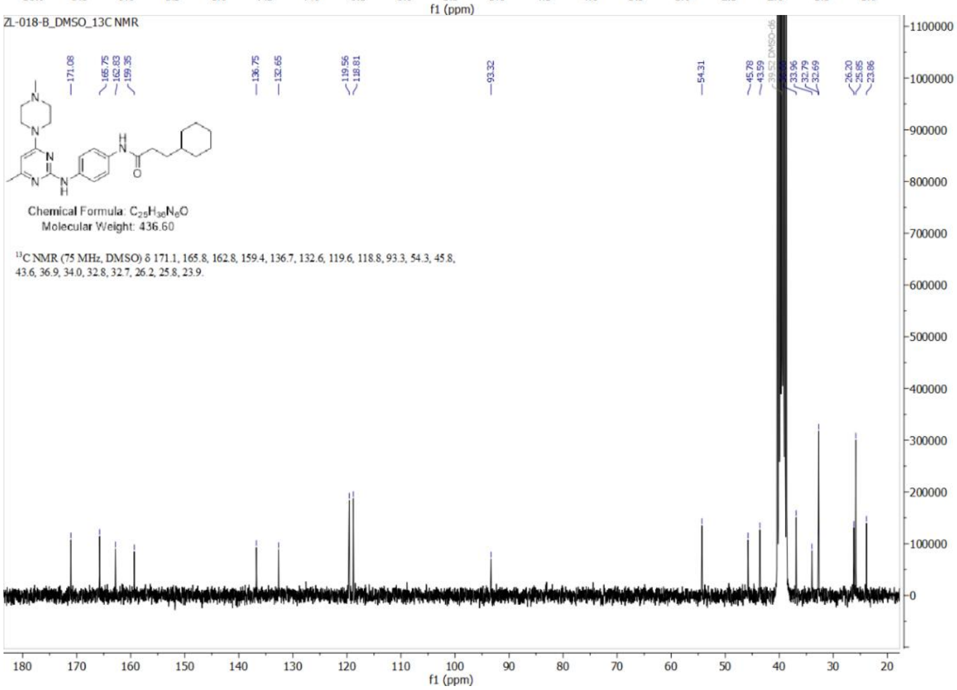

Compound **16**

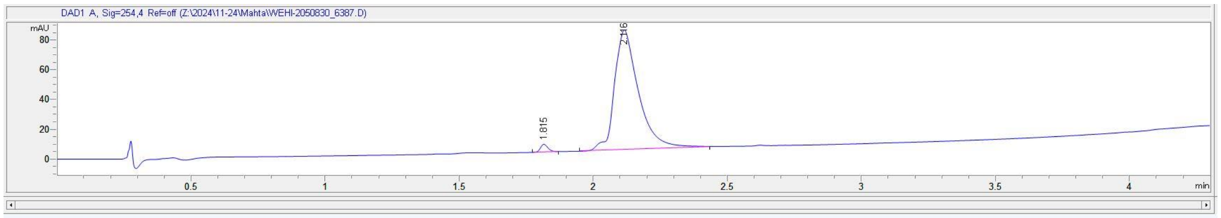

#	Time	Area	Height	Width	Area%	Symmetry
1	1.815	10.3	5.2	0.0304	2.020	0.781
2	2.116	498	80.3	0.0939	97.980	0.655

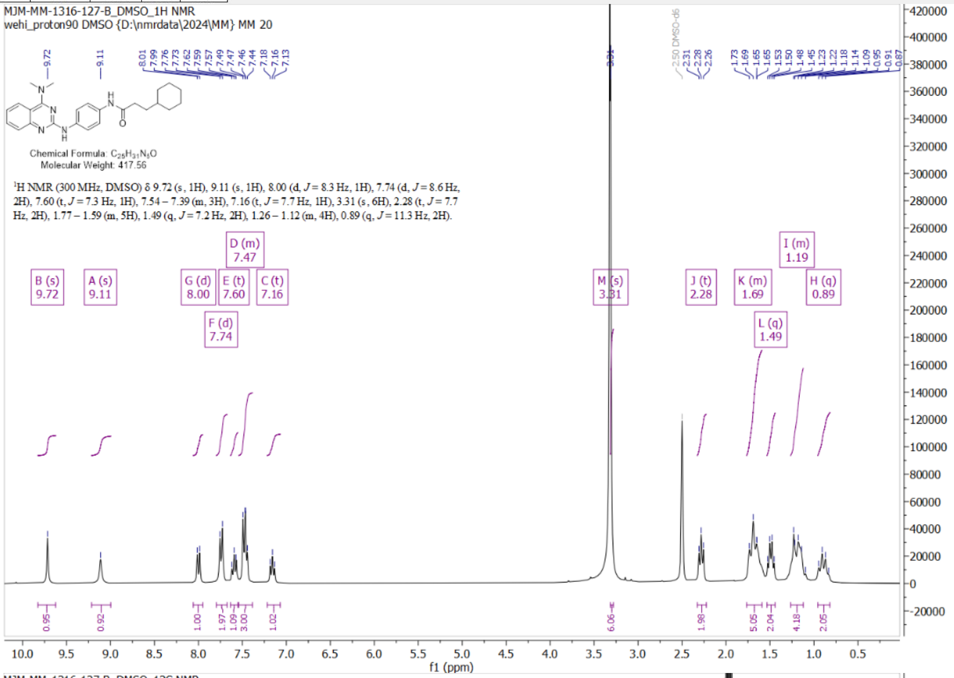

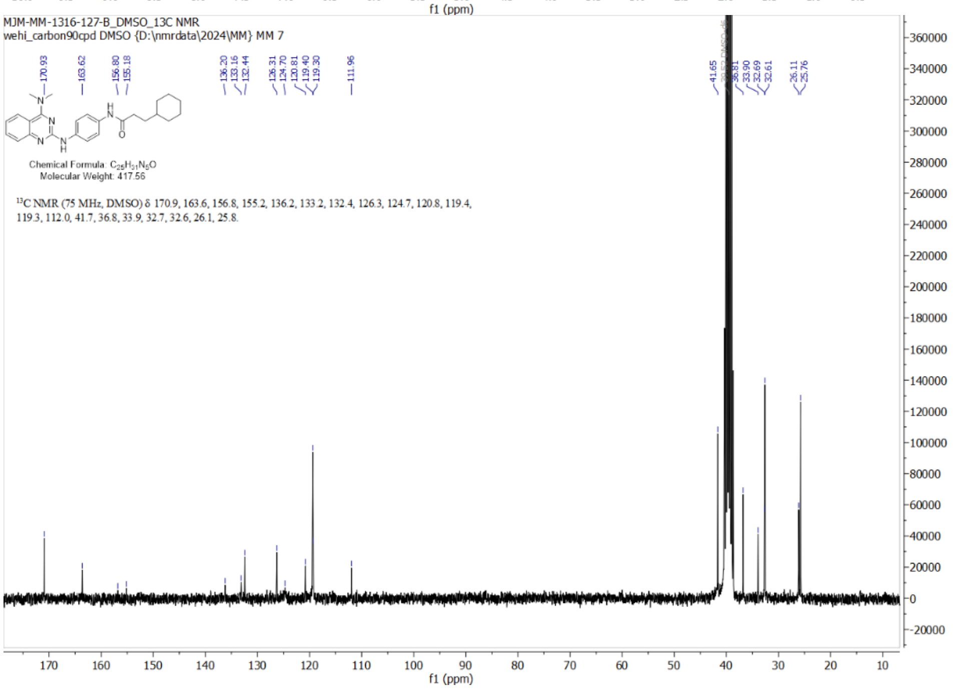

Compound **26**

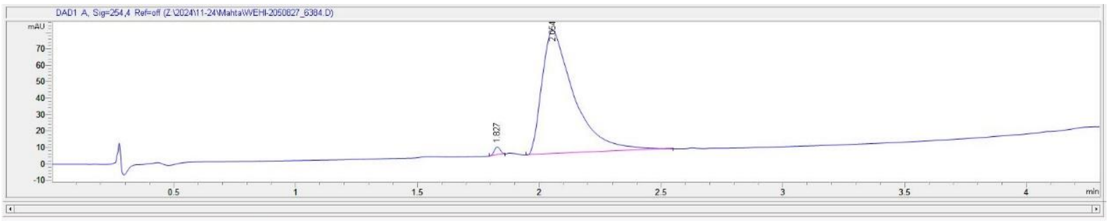

#	Time	Area	Height	Width	Area%	Symmetry
1	1.827	8.4	4.9	0.0275	1.197	0.96
2	2.054	694	75.5	0.1293	98.803	0.447

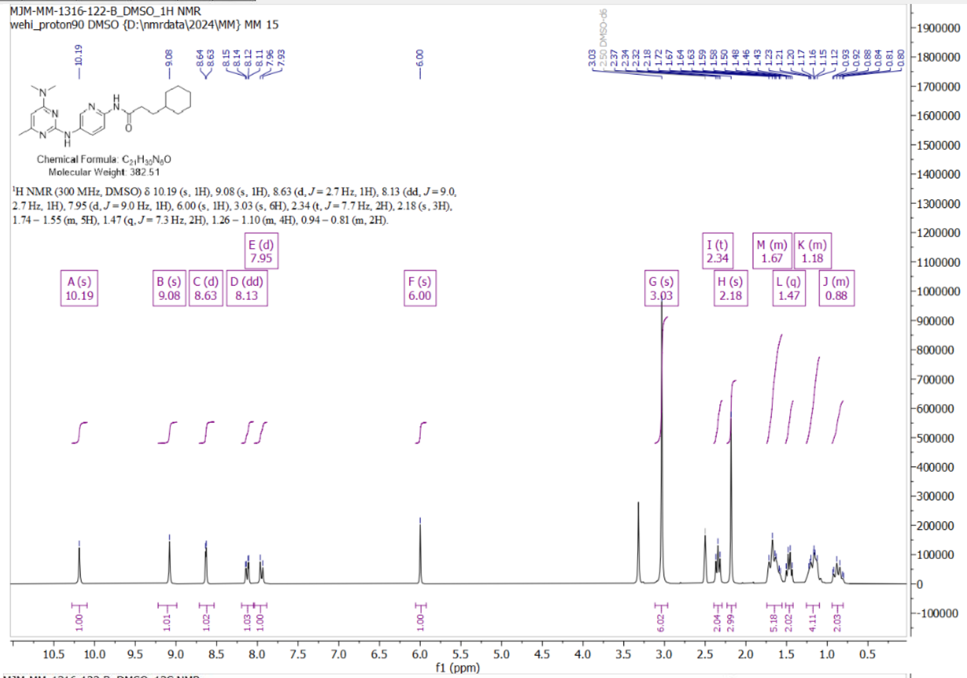

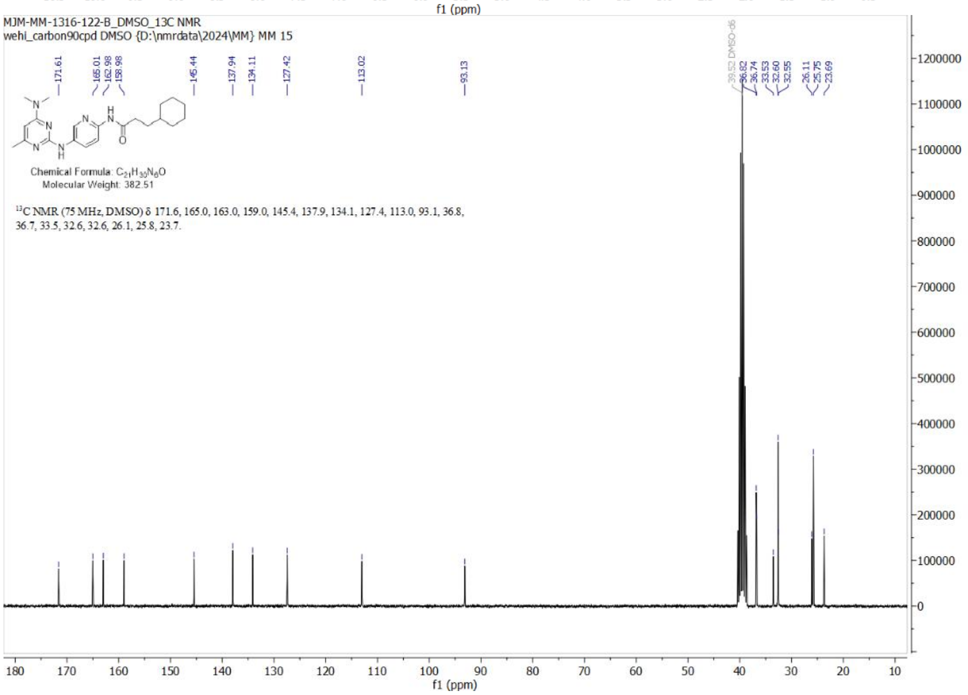

Compound **55**

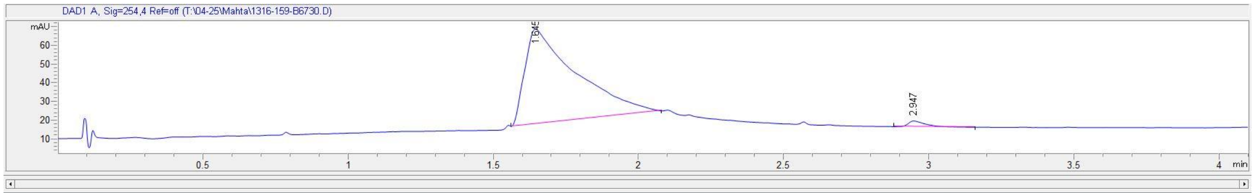

#	Time	Area	Height	Width	Area%	Symmetry
1	1.645	626.4	51.1	0.158	97.976	0.252
2	2.947	12.9	3.1	0.0585	2.024	0.437

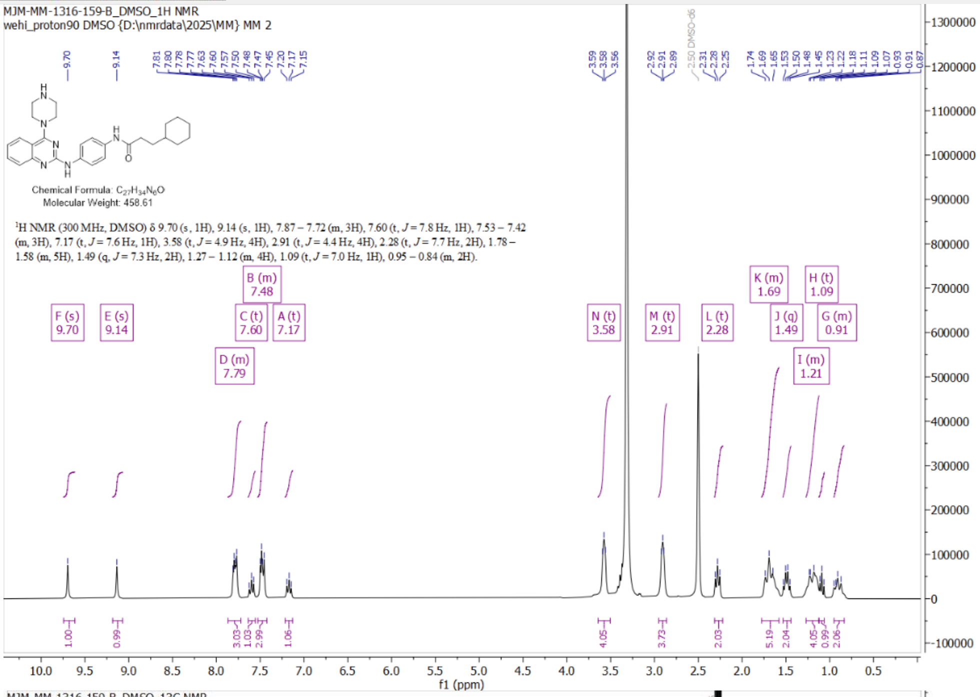

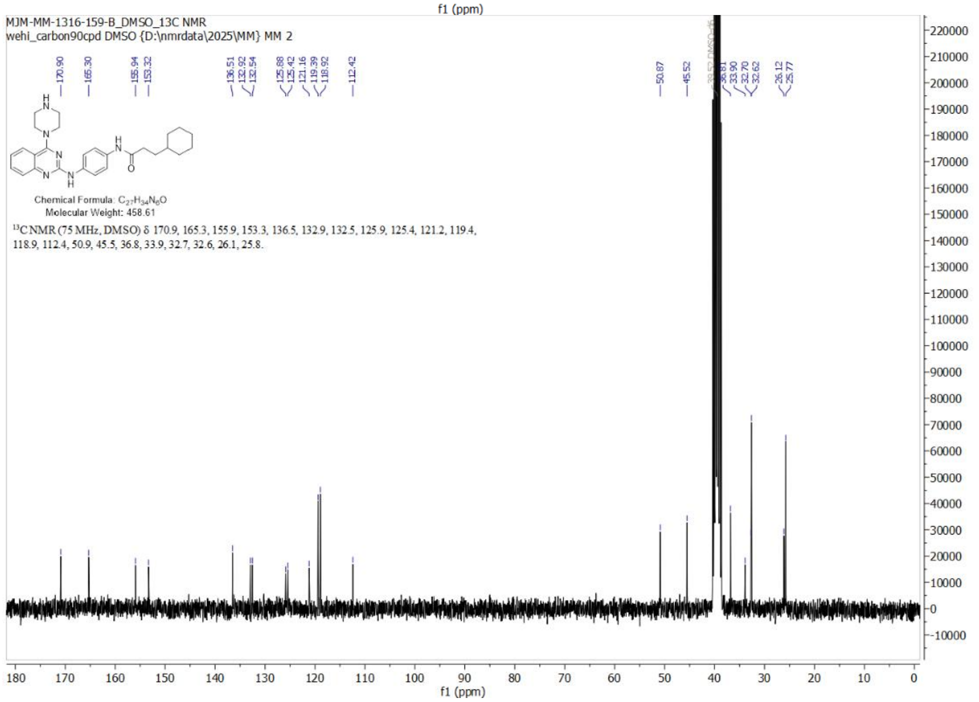

Compound **56**

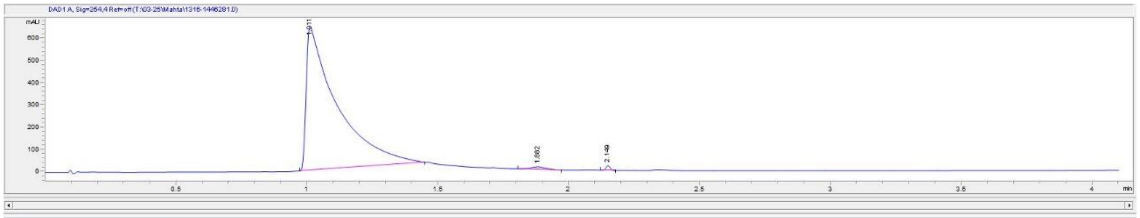

#	Time	Area	Height	Width	Area%	Symmetry
1	1.011	5294.2	650.4	0.103	98.799	0.131
2	1.882	42.4	11.3	0.0567	0.791	0.905
3	2.149	22	20.6	0.0164	0.410	0.954

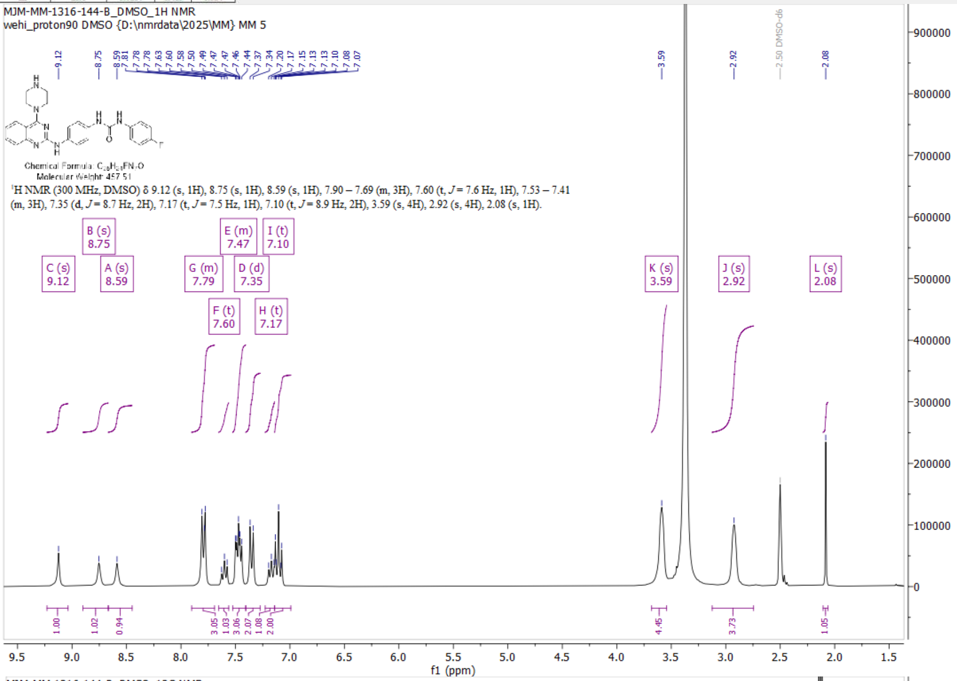

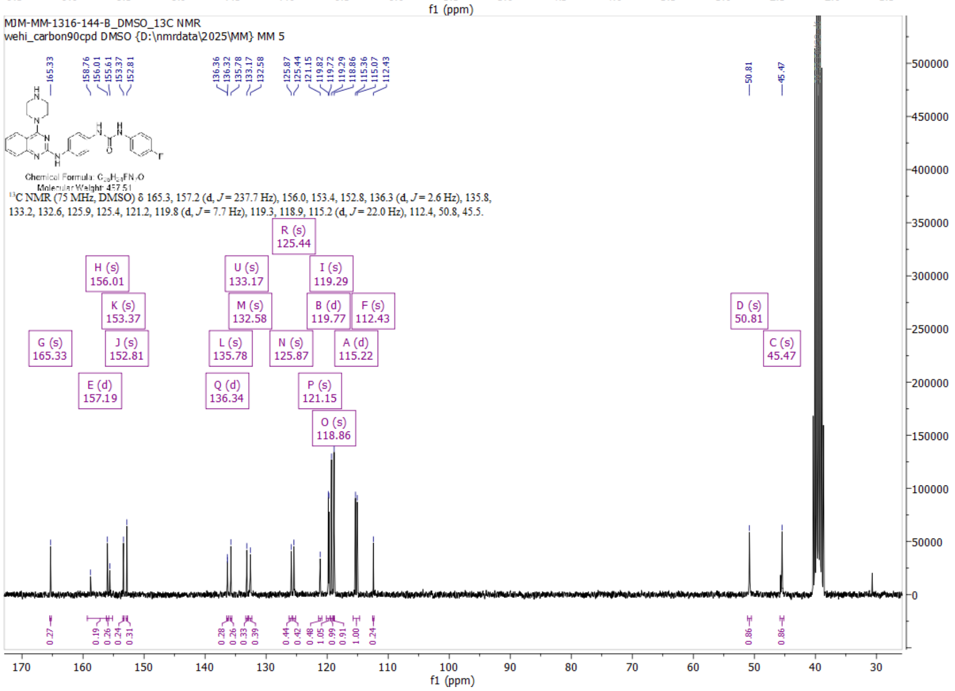

Supplement: Supplementary file 1 — Supplementary Material [file CMDC-21-e202500739-s001.pdf]
